# Absence of Maternal Methylation in Biparental Hydatidiform Moles from Women with *NLRP7* Maternal-Effect Mutations Reveals Widespread Placenta-Specific Imprinting

**DOI:** 10.1371/journal.pgen.1005644

**Published:** 2015-11-06

**Authors:** Marta Sanchez-Delgado, Alejandro Martin-Trujillo, Chiharu Tayama, Enrique Vidal, Manel Esteller, Isabel Iglesias-Platas, Nandita Deo, Olivia Barney, Ken Maclean, Kenichiro Hata, Kazuhiko Nakabayashi, Rosemary Fisher, David Monk

**Affiliations:** 1 Imprinting and Cancer Group, Cancer Epigenetic and Biology Program, Institut d’Investigació Biomedica de Bellvitge, Hospital Duran i Reynals, Barcelona, Spain; 2 Department of Maternal-Fetal Biology, National Research Institute for Child Health and Development, Tokyo, Japan; 3 Cancer Epigenetics Group, Cancer Epigenetic and Biology Program, Institut d’Investigació Biomedica de Bellvitge, Hospital Duran i Reynals, Barcelona, Spain; 4 Department of Physiological Sciences II, School of Medicine, University of Barcelona, Barcelona, Spain; 5 Institucio Catalana de Recerca i Estudis Avançats, Barcelona, Spain; 6 Servicio de Neonatología, Hospital Sant Joan de Déu, Fundació Sant Joan de Déu, Barcelona, Spain; 7 Whipps Cross University Hospital, Barts Health NHS Trust, Leytonstone, London, United Kingdom; 8 Leicester Royal Infirmary, Leicester, United Kingdom; 9 Primary Care Genetics, Sydney, Australia; 10 Imperial Centre for Translational and Experimental Medicine, Imperial College London, London, United Kingdom; 11 Trophoblastic Tumour Screening and Treatment Centre, Department of Oncology, Imperial College London, London, United Kingdom; Aachen Institute of Human Genetics, UNITED STATES

## Abstract

Familial recurrent hydatidiform mole (RHM) is a maternal-effect autosomal recessive disorder usually associated with mutations of the *NLRP7* gene. It is characterized by HM with excessive trophoblastic proliferation, which mimics the appearance of androgenetic molar conceptuses despite their diploid biparental constitution. It has been proposed that the phenotypes of both types of mole are associated with aberrant genomic imprinting. However no systematic analyses for imprinting defects have been reported. Here, we present the genome-wide methylation profiles of both spontaneous androgenetic and biparental *NLRP7* defective molar tissues. We observe total paternalization of all ubiquitous and placenta-specific differentially methylated regions (DMRs) in four androgenetic moles; namely gain of methylation at paternally methylated loci and absence of methylation at maternally methylated regions. The methylation defects observed in five RHM biopsies from *NLRP7* defective patients are restricted to lack-of-methylation at maternal DMRs. Surprisingly RHMs from two sisters with the same missense mutations, as well as consecutive RHMs from one affected female show subtle allelic methylation differences, suggesting inter-RHM variation. These epigenotypes are consistent with *NLRP7* being a maternal-effect gene and involved in imprint acquisition in the oocyte. In addition, bioinformatic screening of the resulting methylation datasets identified over sixty loci with methylation profiles consistent with imprinting in the placenta, of which we confirm 22 as novel maternally methylated loci. These observations strongly suggest that the molar phenotypes are due to defective placenta-specific imprinting and over-expression of paternally expressed transcripts, highlighting that maternal-effect mutations of *NLRP7* are associated with the most severe form of multi-locus imprinting defects in humans.

## Author Summary

Complete hydatidiform moles (CHMs) are abnormal human conceptsus characterized by excessive trophoblast proliferation that commonly result from the absence of a maternal genetic contribution compensated by two copies of the paternal genome. In a few rare cases HMs maybe recurrent (RHM), characterized by a biparental genetic contribution and underlying *NLRP7* mutations. It is speculated that aberrant genomic imprinting plays a key role in HM formation, but to date no studies have determined the extent of imprint defects in molar biopsies. By comparing the methylation profile of CHMs and RHMs with normal placentas, we confirm widespread absence of allelic methylation at imprinted loci and identify many aberrantly methylated regions, all of which have profiles consistent with imprinting.

## Introduction

The most common form of complete hydatidiform mole (CHM) is sporadic and androgenetic diploid in origin. These products of conception frequently result from the fertilization of an oocyte from which the maternal chromosomes are lost and endoreduplication of a single sperm genome, or the fertilization by two sperm, to give a diploid DNA content of entirely paternal origin [1]. Occasionally HM can be recurrent and familial in nature (OMIM 231090) [2]. Detailed homozygosity mapping and gene mutation screening has identified two loci, 19q13.4 and 6q13, which harbor the causative genes, *NLRP7* (NACHT, leucine rich repeat and PYD containing 7) and *KHDC3L* (also known as *C6ORF221*) respectively [3, 4]. Approximately 70% of women affected by familial recurrent HM (RHM) are associated with recessive mutations of *NLRP7* [5, 6], whereas genetic aberrations of *KHDC3L* are much less frequent, and present in only ~10% of patients without an *NLRP7* involvement [7, 8]. In both cases the mutations cause the RHM by maternal-effect. Definitive evidence that a defective oocyte is responsible for the pathophysiology of RHM comes from the observations that assisted reproductive cycles using donated oocytes in three patients with recessive *NLRP7* mutations resulted in normal offspring [8, 9]. This maternal-effect model is consistent with transcript abundance of both *NLRP7* and *KHDC3L*, which accumulate in the developing oocytes and are present in early pre-implantation embryos [10, 11]. Such expression profiles are coherent with an involvement in the control of maternally derived epigenetic programing or early developmental events in the zygote that occur before embryonic genome activation. Paternal transmission of *NLRP7* mutations does not interfere with spermatogenesis, since males homozygous for *NLRP7* mutations can father children [5, 12].

Epigenetic studies in these abnormal pregnancies have revealed aberrant DNA methylation profiles at a limited number of imprinted genes [13, 14]. Imprinted genes are expressed in a parent-of-origin specific fashion, which is coordinated by differentially methylation regions (DMRs) inherited from the gametes [15]. *NLRP7* does not have an orthologue in mouse, but is thought to have originated from an evolutionary duplication of its nearest family member, *NLRP2* [16]. Curiously, mutations of *NLRP2* are responsible for a single familial case of Beckwith-Wiedemann syndrome with methylation defects at multiple loci, including *Kv*DMR1 (also known as ICR2) and *MEST* DMR [17].

Consistent with their androgenetic composition, our recent genome-wide methylation profiling of sporadic HMs revealed the paternalization of all known imprinted DMRs, with maternally-methylated DMRs being devoid of methylation and paternally-derived DMRs being fully methylated [18]. Similar analyses on RHM biopsies tissues with known underlying genetic causes are difficult to conduct, partially hampered by the fact that genetic diagnosis takes place in the phenotypically normal affected women, with the molar tissues discarded following pathological examination.

## Results

### DNA methylation profiling of ubiquitous imprinted DMRs in *NLRP7* mutated RHMs

**Fig 1:**
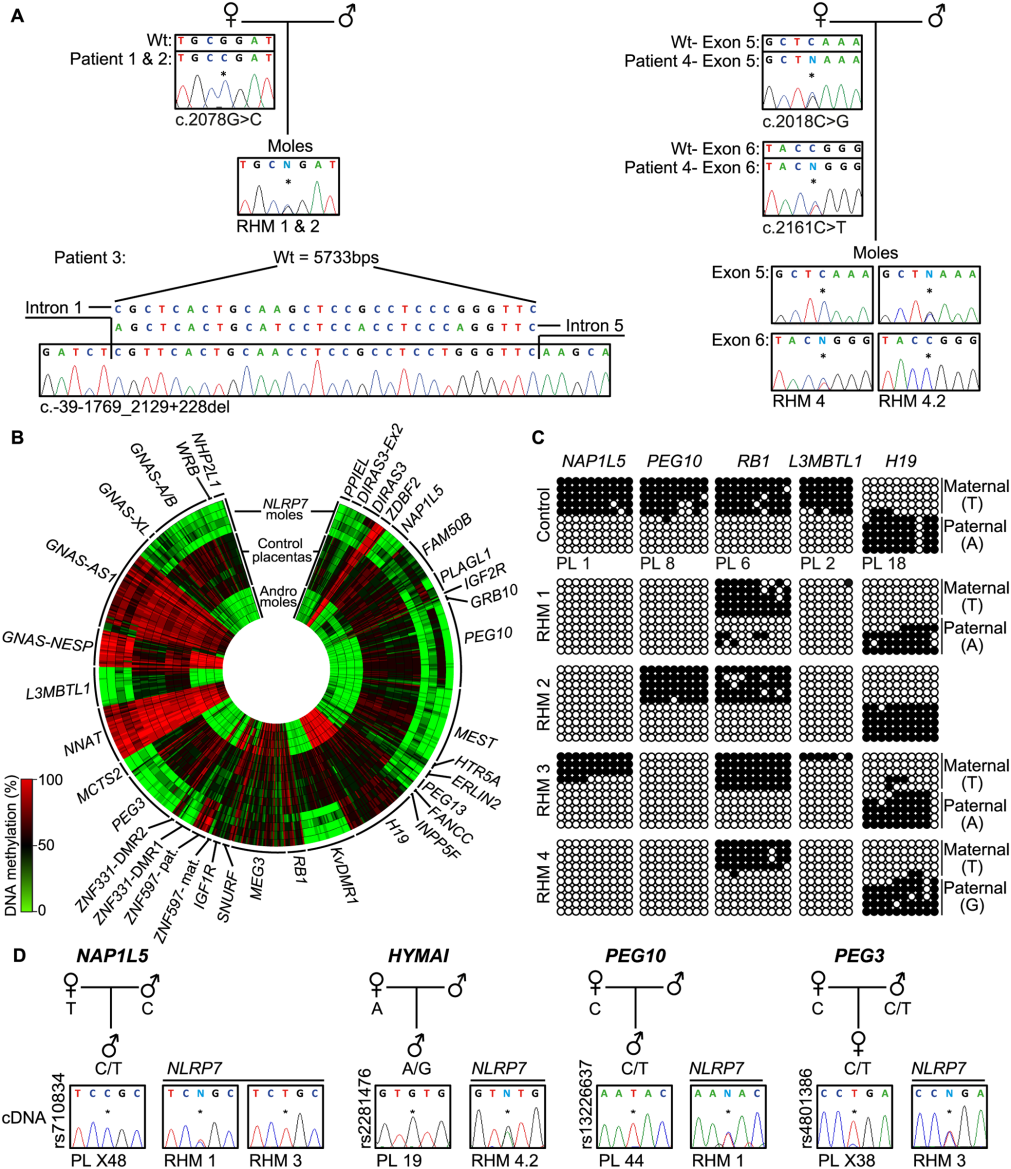
Description of *NLRP7* mutations with methylation and expression profiling of imprinted loci. (A) Confirmation of recessive *NLRP7* mutations in female patients and heterozygous status in the RHM samples. The asterisk (*) on the electropherogram highlights the position of the mutation. For patient 3 the position of the deletion is shown. (B) Circular heat map of the 616 Infinium array probes mapping to 36 ubiquitously imprinted DMRs. The inner circle represents the methylation values of androgenetic HMs, the middle circles normal placental biopsies and the outer circle the RHMs associated with maternal-effect *NLRP7* mutations. (C) Confirmation of the methylation profile of the *NLRP7* mutated RHMs at the *NAP1L5*, *PEG10*, *RB1*, *L3MBTL1* and *H19* DMRs by bisulphite PCR and subcloning. Each circle represents a single CpG dinucleotide on a DNA strand, a methylated cytosine (●) or an unmethylated cytosine (○). For clarity, only the first 10 CpG dinucleotides from each amplicon are shown with the letters in the parentheses indicating SNP genotype. (D) Allelic expression analysis of imprinted genes *NAP1L5*, *HYMAI*, *PEG10* and *PEG3* in control placenta samples (PL) and *NLRP7*-mutated moles (RHM).

In this study, we determine the genome-wide methylation profiles of RHMs from four females with *NLRP7* mutations using the high-density Illumina Infinium HumanMethylation450 (HM450k) BeadChip arrays, which simultaneously quantify methylation at ~2% of all CpG dinucleotides in the human genome. The RHM samples were from women with a variety of genetic lesions including siblings carrying the same homozygous non-synonymous missense mutation (c. 2078G>C; p.R693P), an individual homozygous for a deletion that removes exons 2–5 (c.-39-1769_2129+228del) and a female with compound heterozygous mutations (c.2018C>G, c.2161C>T; p.S673X, p.R721W) (Fig 1A). Our initial analysis focused on comparing the methylation profiles of four androgenetic moles and seven normal placental samples (three first trimester and four third trimester) with those obtained for the *NLRP7*-mutated samples (Fig 1B and S1A Fig). A total of 616 probes mapping to 36 known ubiquitous imprinted DMRs were assessed, with observations confirmed by both pyrosequencing and standard allele-specific bisulphite PCR and sub-cloning (Fig 1C, S1C and S2 Figs). These comprehensive analyses revealed that, while normal placental biopsies had partial methylation consistent with allelic methylation (with the exception of the fully methylated *NNAT* and *GNAS*-AS1 promoters) [18], the majority of maternally methylated DMRs presented with lack-of-methylation (LOM) in both androgenetic and *NLRP7*-associated HMs. The only exceptions were for the *IGF1R* and *RB1* DMRs that maintain allelic methylation in both types of mole, whereas the *SNURF* DMR was maintained in some of the *NLRP7*-mutated samples. In addition, we observe some inter-individual differences. The *FAM50B* DMR maintained a partially methylated state in two androgenetic CHMs and in a RHM from one of the sisters with the *NLRP7* p.R693P mutation. Surprisingly, this same RHM sample also showed imprinted methylation at the *PLAGL1* and *PEG10* DMRs (Fig 1C and S2 Fig). Furthermore a comparison of two different RHMs from patient 4 revealed a similar methylation profile with the exception that the *PEG10* and *SNURF* DMRs presented allelic methylation in one of the moles (S1 Fig). The *PEG10* DMR was previously reported to be largely unaffected in three familial RHM samples [14]. The only paternal DMR with probes present on this array that acquires methylation in the male germline and is partially methylated in placenta is the *H19* DMR (also known as ICR1). Consistent with the two copies of the sperm genome, the androgenetic CHMs are fully methylated at this locus, whereas the RHM are partially methylated. In 3 cases allele-specific bisulphite PCR revealed that the methylation was on the paternal allele (Fig 1C). Quantitative pyrosequencing of bisulphite PCRs targeting the IG-DMR on chromosome 14, which also acquires methylation from sperm but does not have probes on the HM450k array platform, revealed a partially methylated profile in both control placenta and RHMs (S2 Fig). Similarly the *MEG3* DMR, which is regulated in-*cis* by the IG-DMR, shows a similar partially methylated profile consistent with allelic methylation (Fig 1B). The *ZBDF2* and *ZNF597/NAA60* promoters were fully methylated in both androgenetic CHM and *NLRP7* mutated RHMs. This is consistent with the presumption that these regions acquire methylation on the paternal allele during early development under the hierarchical influence of the maternally methylated *GPR1*-AS and *ZNF597* DMRs, respectively [18].

To determine if the lack of methylation at imprinted DMRs results in altered expression we performed allelic-specific RT-PCR on the RHM samples. We confirm that *HYMAI*, *PEG10* and *PEG3* transcripts are paternally expressed in control placenta samples but expressed from both alleles in RHMs (Fig 1D). Biallelic expression of *NAP1L5* was associated with LOM in RHM1, but imprinted expression was preserved in RHM3 that had allelic methylation at this DMR (Fig 1C and 1D).

### Widespread absence-of-methylation at placenta-specific imprinted loci

**Fig 2:**
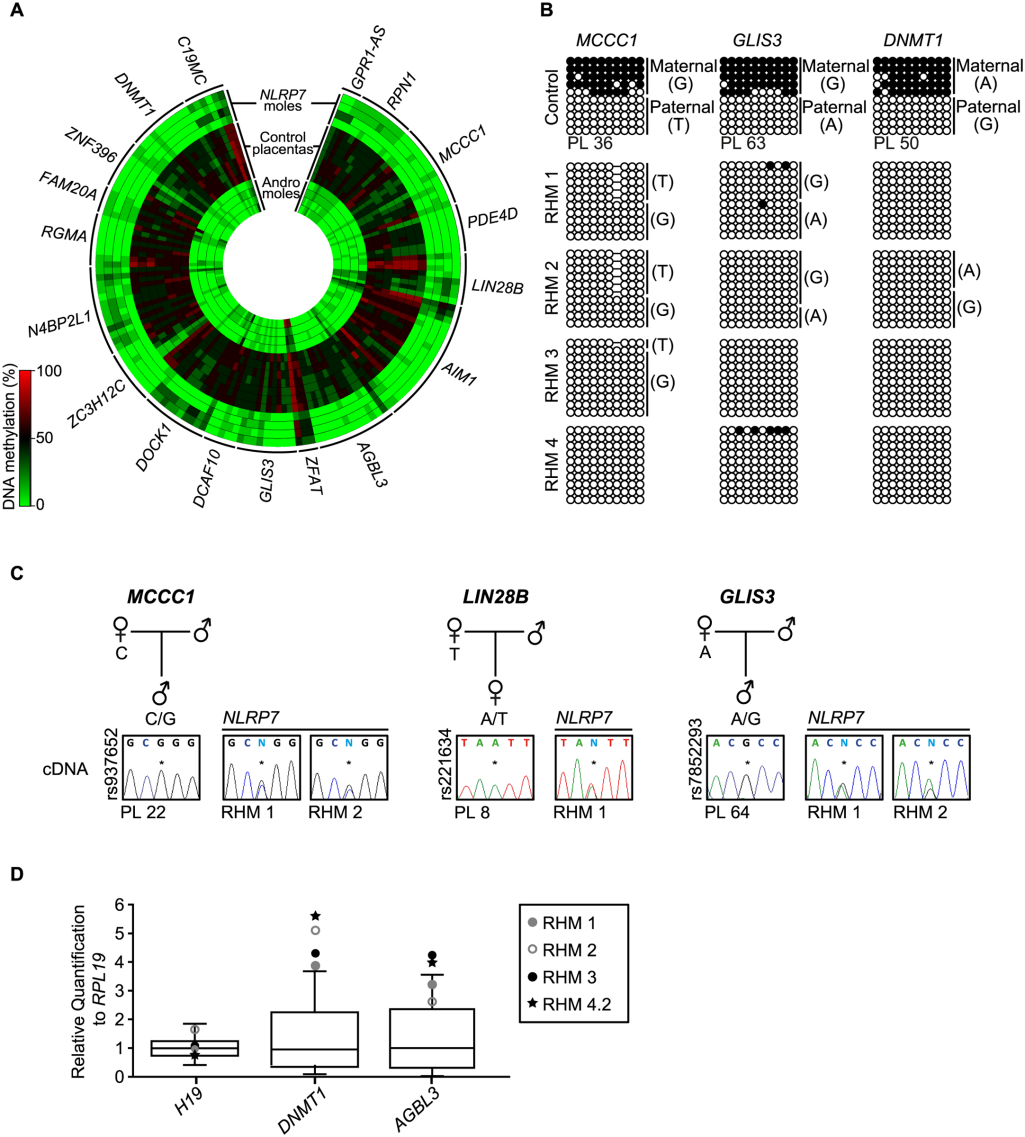
Methylation and expression analyses of placenta-specific DMRs in RHM samples. (A) Circular heatmap of the 153 Infinium array probes mapping to the 18 known placenta-specific imprinted DMRs. The inner circles represent the methylation values of androgenetic HMs, the middle circles normal placental biopsies and the outer circle the RHMs associated with maternal-effect *NLRP7* mutations. (B) Confirmation of the methylation profile at the maternally methylated *GLIS3*, *DNMT1* and *MCCC1* DMRs by bisulphite PCR and subcloning. Each circle represents a single CpG dinucleotide on a DNA strand, a methylated cytosine (●) or an unmethylated cytosine (○). For clarity, only the first 10 CpG dinucleotides from each amplicon are shown with the letters in the parentheses indicating SNP genotype. (C) Allelic expression analysis of imprinted genes *MCCC1*, *LIN28B* and *GLIS3* in control placenta samples (PL) and *NLRP7*-mutated moles (RHM). (D) Quantitative RT-PCR for *H19*, *DNMT1* and *AGBL3* in RHM samples. The boxplot show the median expression (whiskers 5–95% percentile) determined for 15 control placenta samples with the values of RHMs highlighted.

Recently, using genome-wide methylation profiling in normal placental biopsies and androgenetic moles, we identified 18 placenta-specific maternally methylated DMRs [18]. Genome-wide methylation analysis utilizing methyl-seq in human gametes revealed that these loci inherit methylation from oocytes and maintain allelic methylation during pre-implantation reprograming [19]. We interrogated the 153 probes mapping to these placenta-specific imprinted DMRs and confirmed observations by both pyrosequencing and allele-specific bisulphite PCR (Fig 2, S1 and S2 Figs). This revealed that, while first trimester and term placental biopsies had partial methylation indicative of maternally methylated DMRs, all androgenetic and *NLRP7*-associated HMs presented with robust LOM. In several cases the RHM samples were heterozygous for single base pair polymorphisms (SNPs) that confirmed that methylation was absent from the maternal alleles (Fig 2B). Consistent with the lack of allelic methylation, the normally paternally expressed imprinted genes *MCCC1*, *LIN28B* and *GLIS3* are expressed from both parental alleles in RHMs (Fig 2C). Furthermore qRT-PCR revealed an increased expression of *DNMT1* and *AGBL3* compared to normal placenta samples coherent with biallelic over-expression. Expression was within the normal range for *H19* which in consistent with the maintained paternally derived methylation at this DMR (Fig 2D). Furthermore, our previous results revealed that approximately 12% of all CpG methylation is contained within *LINE*-1 sequences [20]. Pyrosequencing analysis of these retrotransposable elements, as well as α-satellites and Alu-Yb8 sequences, in *NLRP7*-mutated RHMs revealed a profile indistinguishable from normal placenta (S3 Fig) [18]. Together these observations suggest that only maternally derived methylation is affected in RHM, consistent with oocyte epigenetic aberration and not somatic imprint maintenance.

### Identification of novel maternal methylated regions in human placenta

**Fig 3:**
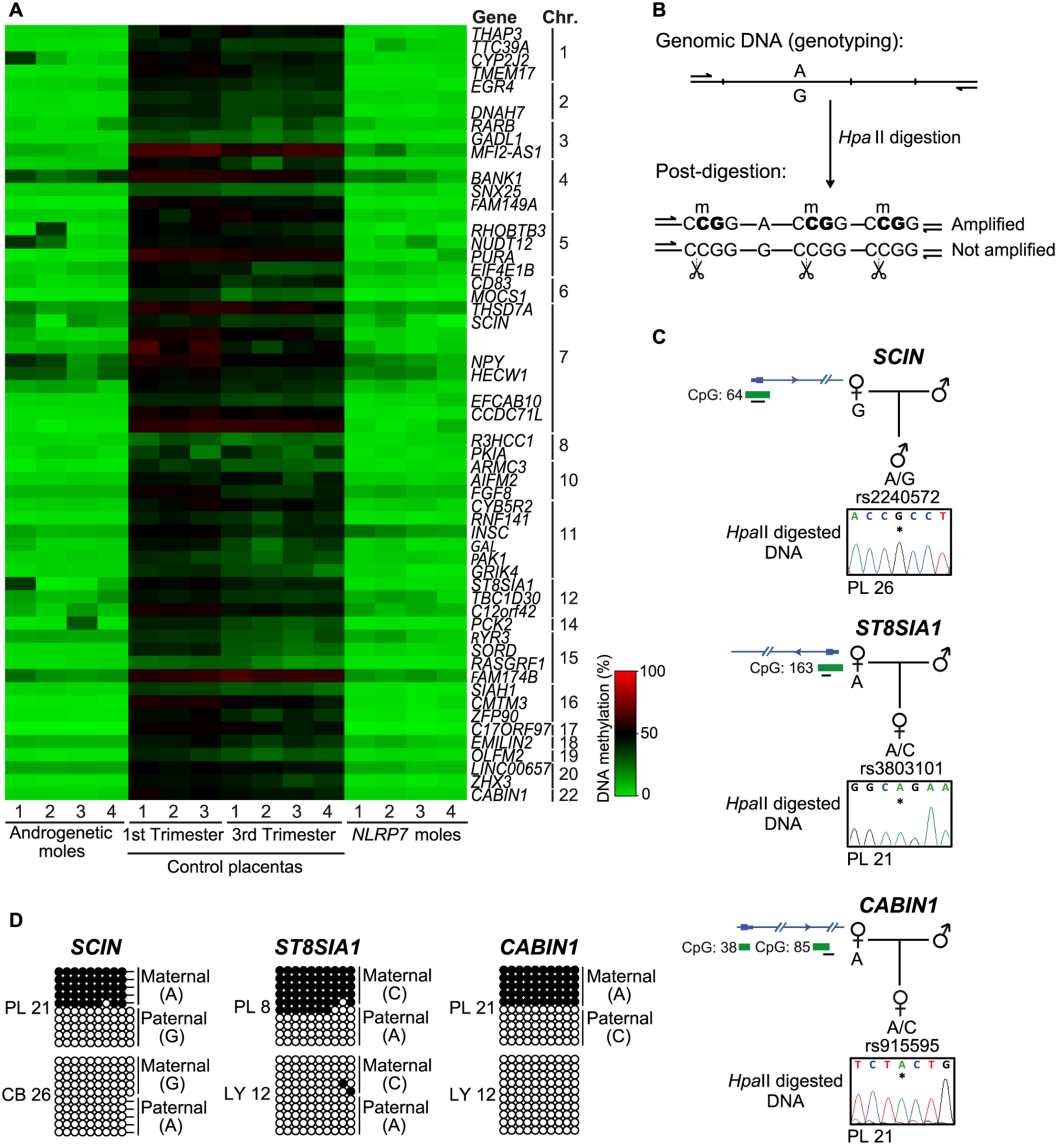
Identification of additional placenta-specific imprinted DMRs in RHM samples. (A) A heatmap for the β_mean_ of the Infinium probes with a methylation difference (>20%, minimum 3 consecutive probes) in RHMs associated with maternal effect *NLRP7* mutations compared to control placental biopsies. (B) Schematic representation of the methylation-sensitive *Hpa*II genotyping assay. (C) Methylation profiles as determined by methylation-sensitive genotyping and (D) bisulfite PCR and subcloning on placenta and somatic tissue DNA samples at the *SCIN*, *ST8AIA1* and *CABIN1* promoters. Note that the samples used for methylation-sensitive genotyping and bisulphite PCR maybe different to highlight that methylation is not associated with genotype but parental origin.

To expand our methylation analysis we performed an unbiased screen for additional loci with abnormal methylation in the RHM samples with underlying *NLRP7* mutations. The identified regions were characterized by at least 3 Infinium probes and relative distance between consecutive probes below 500 bp, requiring runs with a consistent change (same direction and p-value < 0.01) and an absolute average methylation change >20% (β 0.2). This analysis identified 61 regions, 56 of which are CpG islands, 88% mapping to transcript promoters (Fig 3A; S1 Table). Surprisingly all candidate regions identified were partially methylated in normal placental biopsies and devoid of methylation in androgenetic CHMs and somatic tissues (Fig 3A; S1 Table). This profile suggests the existence of further placenta-specific maternally methylated regions that could regulate imprinted expression. Consistent with the regions being maternally methylated, all regions were unmethylated in sperm (S1 Table). To confirm if the observed methylation was restricted to the maternal allele we developed a methylation-sensitive genotyping assay in which polymorphic allele calling is performed on genomic DNA before and following digestion with the methylation-sensitive *Hpa*II endonuclease (Fig 3B). Allelic methylation is confirmed when a heterozygous genomic DNA sample is reduced to homozygosity following digestion with the remaining allele representing the methylated chromosome. Twenty-eight of the 61 candidate regions had highly informative SNPs that allowed parental origin of methylation to be determined. In 22 cases we confirmed the presence of maternal methylation in multiple placenta samples, with a further six regions being allelically methylated with parental genotypes being uninformative (Fig 3C and 3D, S4 and S5 Figs; S2 Table). Fifteen of these samples were subsequently shown to be allelically methylated using bisulphite PCR and subcloning with an additional five regions associated with *RHOBTB3*, *PURA*, *FGF8*, *CCDC71L* and *WIF1* presenting with both fully methylated and unmethylated DNA strands (Fig 3D and S5 Fig).

### Allelic expression analysis reveals additional imprinted genes in the human placenta

**Fig 4:**
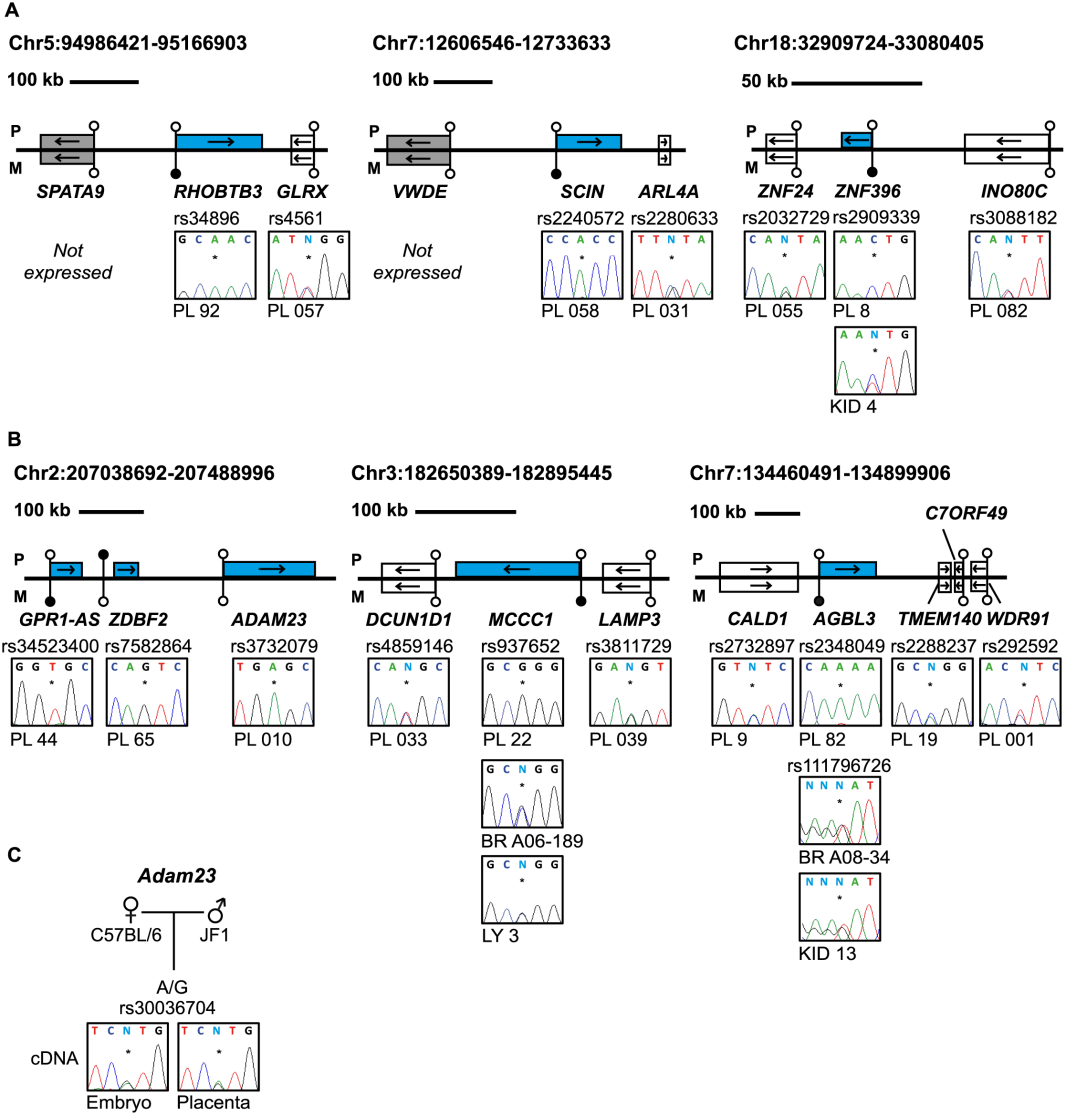
Allele-specific RT-PCR analysis of candidate placenta-specific imprinted genes. (A) Confirmation of paternal expression of *RHOBTB3*, *SCIN* and *ZNF396* in term placenta and biallelic expression of neighboring genes. (B) The allele-specific expression analysis of genes flanking known placenta-specific imprinted transcripts *GPR1-AS*, *MCCC1* and *AGBL3*. Biallelic expression of *ZNF396*, *ADAM23*, *MCCC1* and *AGBL3* was confirmed in somatic tissues. (C) Allele-specific RT-PCR analysis of *Adam23* in mouse embryo and placenta at embryonic day 9.5. The asterisk (*) in the sequence traces shows the position of the polymorphic base. The blue boxes in the figures represent the paternally expressed transcripts, white boxes signify biallelically expressed genes and grey boxes are transcripts not expressed in term placenta samples. The location of unmethylated CpG islands and the DMRs are shown by the lollipops. PL = placenta, BR = brain, KID = kidney, LY = blood leucocytes, CB = cord blood.

The main biological significance of allele-specific methylation is allele-specific RNA expression, which in the case of maternally methylated regions is predicted to dictate paternal expression. We subsequently determined allelic expression for a subset of transcripts that contained highly polymorphic exonic SNPs. Allele-specific RT-PCR confirmed paternal expression of *RHOBTB3*, *SCIN*, *ZNF396*, *ST8SIA1*, *ZFP90*, *CCDC71L*, *RASGRF1*, *HECW1* and *CMTM3* with monoallelic expression of *CD83* in a polymorphic fashion in multiple placental biopsies (Fig 4A and S6A Fig; S3 Table). In situations where monoallelic expression was uninformative due to maternal DNA also being heterozygous, the methylated allele was always the repressed one, suggesting a functional link between methylation and expression. In addition, we identified a maternally methylated CpG islands overlapping the promoter of *SNCB*, a transcript that has previously been described as paternally expressed in placenta [21]. Furthermore, we also identified a maternal DMR within the *TTC39A* gene that is adjacent to *EPS15*, a transcript also reported to be imprinted in placenta [22] (S5 Fig).

To determine whether these placenta-specific DMRs can orchestrate allelic silencing of gene clusters similar to ubiquitous imprinted DMRs, we performed allele-specific RT-PCR for 20 flanking genes associated with loci containing imprinted transcripts (Fig 4B and S6B Fig). Surprisingly, with the exception of *ADAM23* within the *GPR1-AS* domain on chromosome 2, we observe that the remaining 19 transcripts analyzed are expressed equally from both parental chromosomes, indicating that placenta-specific DMRs do not possess the ability to regulate allelic expression of surrounding genes. Despite evolutionary conserved imprinting of *GPR1-AS* and *ZDBF2* [23], paternal expression of *ADAM23* is not observed in mouse placenta (Fig 4C). Together this suggests that this locus is regulated in a different manner to the majority of placenta-specific imprinted loci identified and that subtle species differences exist [24].

## Discussion

We have compared the DNA methylation profiles of RHMs with underlying *NLRP7* mutations with androgenetic CHM biopsies. This has revealed not only widespread methylation defects at imprinted loci, but has facilitated in the identification of novel maternally methylated loci. As a result of unbiased bioinformatic analyses, the number of genes associated with placenta-specific maternally methylated DMRs has increased from 18 to 43, with a further eight regions of allelic methylation indicative of an imprinted DMR. In addition, a further 28 candidates did not contain SNP allowing allelic methylation to be determined. Our observations indicate that there are more imprinted domains in the human placenta than in somatic tissues. Interestingly, 10 of these loci (*TMEM17*, *NUDT12*, *RHOBTB3*, *CD83*, *ARMC3*, *AIFM2*, *ST8SIA1*, *PCK2*, *RASGRF1*, *CMTM3*) show opposing methylation profiles in diandric (two paternal plus one maternal haploid genomes) compared to digynic (extra maternal chromosomes) triploid biopsies [25], consistent with the maternal methylation profile we describe.

It is currently unknown which imprinted genes are responsible for the HM phenotype, but aberrant expression from the maternal allele of these placenta-specific genes is likely to play an important role, since they include the essential epigenetic gene *DNMT1* and the micro-RNA processor *LIN28B*. In addition there are several strong candidates for influencing trophoblast development, including the cytochrome P450 (CYP) subfamily member *CYP2J2* that has previously been shown to be up-regulated in preeclampsia and *THSD7A*, a placental and endothelial protein that mediates cellular migration [26, 27]. In addition, the deregulation and over-expression of the C19MC pri-miRNA will lead to the concomitant increased abundance of 50 mature miRNAs that have recently been shown to regulate trophoblast invasion [28, 29].

Our genome-wide analysis revealed aberrant methylation profiles in RHM associated with maternal-effect *NLRP7* defects at imprinted loci. It is not possible to determine if the methylation anomalies are restricted to imprinted loci, as many regions inheriting methylation from oocytes undergo epigenetic reprograming during pre-implantation development [30, 31], resulting in an unmethylated state that is indistinguishable from an epimutation. Similar epigenetic profiling of blood-derived DNA from the women carrying biallelic recessive genetic mutations of *NLRP7* failed to identify any methylation anomalies when compared to healthy controls (S7 Fig), endorsing the hypothesis that the methylation defect arises by maternal-effect either in the developing oocyte or in early pre-implantation stages. Our results imply that the epigenetic aberration observed in RHM arise early in the female germline since paternally methylated DMRs are unaffected, maintaining the correct methylation profiles at the *H19* and IG-DMR loci. This suggests that NLRP7 has a different function to ZFP57 [32] or DPPA3 [33], which both protect multiple imprints from TET3-associated 5mC to 5hmC reprogramming at the zygotic stage when the pronuclei have yet to breakdown and the parental genomes fuse [34]. Endorsing this theory, detailed immunostaining for NLRP7 in early human embryos revealed that this protein is exclusively localized to the cytosekeleton and not in the nucleus where it could associate with chromatin and influence methylation [11]. In addition, NLRP7 is not observed in the nucleus of developing oocytes (germinal vesicle (GV) stage oocytes through to those arrested in metaphase II) [5, 11]. Curiously, immunostaining for the *de novo* methylatransferases DNMT3A and DNMT3B revealed a similar cytoplasmic localization [35], indicating that a NLRP7-complex may ensure the correct cellular localization and nuclear translocation of these epigenetic factors during a yet to be identified period of oocyte development. Once in the nucleus, this low abundance complex may associate to specific DNA sequences by direct interaction with chromatin regulator YY1 or ZBTB16 [36, 37]. This, and the absence of DNMT3L in human GV-metaphase II oocytes, highlights the fact that the process of imprint acquisition in humans and mouse differ greatly.

The NLRP family of proteins is known to play a direct role in inflammasome activation, which results in the secretion of interleukin-1β (IL-1β) [38]. These observations offer an indirect mechanism explaining how NLRP7 influences maternally derived methylation. Prenatal oogenesis produces hundreds of thousands of oocytes, most of which are discarded before birth. During fetal development this phenomenon is associated with oocyte apoptosis, acting as a quality control measure, eliminating cells with meiotic anomalies. Interestingly, strong NLRP7 staining has been reported in human blastomeres undergoing apoptosis [11]. The process of oocyte selection can be influenced by pro-survival factor IL-1β, a cytokine known to be involved in oocyte nuclear maturation in many mammalian species [39]. This reduction in oocyte number occurs at the approximate time (14–20 weeks gestation) when they presumably acquire the methylation signatures at imprinted regions [40]. It is therefore plausible that disruption to the selection mechanism through defective NLRP7 may allow for the survival, and eventual dominant follicle recruitment, ovulation and fertilization decades later, of an oocyte with an inappropriate methylation state. Whatever the underlying mechanism, we show that maternal-effect mutations of *NLRP7* are associated with the most severe cases of multi-locus imprinting defects in humans.

## Material and Methods

### Ethics statement

All women had presented with multiple RHMs (patients 1–4 had 3, 6, 4 and 3 previous RHMs, respectively) and provided informed consent to use their tissues for research. The ethical approval was granted by the Bellvitge Institute for Biomedical Research (PR096/10) and the Tissue Management Committee of the Imperial College Healthcare NHS Trust Research Tissue Bank (R15048), which is approved by NRES to provide deemed ethics for projects accessing material and data stored within the Research Tissue Bank. All mothers provided informed consent for themselves and their child prior to participating in the study. Ethical approval for collecting blood and placental samples was granted by the ethical committees of Hospital St Joan De Deu Ethics Committee (Study number 35/07), Bellvitge Institute for Biomedical Research (PR006/08) and the National Center for Child Health and Development (project 234). Peripheral blood samples were obtained from healthy volunteers and tissue samples were obtained from BrainNet Europe/Barcelona tissue bank. Mouse work was approved by the Institutional Review Board Committees at the National Center for Child Health and Development (approval number A2010-002). Animal husbandry and breeding were conducted according to the institutional guidelines for the care and the use of laboratory animals.

### Patient samples

Five molar biopsies from four different women with two mutated copies of the *NLRP7* gene who were referred to the Trophoblastic Tumour Screening and Treatment Centre, Charing Cross Hospital (London, UK) were used in this study. The mutations were identified using standard PCR and sequencing as previously described [5]. All women had presented with multiple RHMs and provided informed consent to use their tissues for research.

A cohort of 72 human placenta biopsies with corresponding maternal blood samples were collected at Hospital St Joan De Deu (Barcelona, Spain) and the National Center for Child Health and Development (Tokyo, Japan). All placenta biopsies were collected from the fetal side around the cord insertion site. The placenta-derived DNA samples were free of maternal DNA contamination based on microsatellite repeat analysis. Both DNA and RNA extractions and cDNA synthesis were carried out as previously described [20, 41].

### Mouse crosses

Wild type mouse embryos and placentae were produced by crossing C57BL/6 (B) with *Mus musculus molosinus* (JF1) mice and collected at embryonic day 9.5.

### Methylation array hybridisation

We generated methylation datasets using the Illumina Infinium HumanMethylation450 BeadChip arrays, which simultaneously quantifies ~2% of all CpG dinucleotides. Bisulphite conversion of 600 ng of DNA was performed according to the manufacturer’s recommendations for the Illumina Infinium Assay (EZ DNA methylation kit, ZYMO, Orange, CA). The bisulphite-converted DNA was used for hybridisation following the Illumina Infinium HD methylation protocol at genomic facilities of the Cancer Epigenetics and Biology Program (Barcelona, Spain) or the Barts and The London School of Medicine and Dentistry Genome Centre (London, UK). The resulting data for the *NLRP7*-mutated familial RHMs and the corresponding maternal blood samples have been deposited in the GEO database with the accession number GSE66247. In addition we used the androgenetic CHMs, control placenta and leukocyte datasets from GSE52576.

### Data filtering and analysis

Before analysing the data, we excluded possible sources of technical biases that could influence results. We applied signal background subtraction and inter-plate variation was normalized using default control probes in BeadStudio (version 2011.1_Infinium HD). We discarded probes with a detection p-value >0.01. We also excluded probes that lack signal values in one or more of the DNA samples analysed. For the analysis of known imprinted domains, probes mapping to the DMRs identified by Court and colleagues were directly analysed. Prior to screening for novel imprinted DMRs we excluded all X chromosome CpG sites. An in-house bioinformatic pipeline (using R-package) was utilized to tests the difference of a minimum of 3 consecutive Infinium probes within 500bp windows via a linear model (empirical Bayes moderated p-value < 0.01) that provides a t-statistic, with an absolute methylation change of > 20% (beta 0.2). The circular heatmaps used to display the DNA methylation profiles were generated using Circos software.

### Genotyping and imprinting analysis

Genotypes of potential SNPs identified in the UCSC hg19 browser were obtained by PCR and direct sequencing. Sequence traces were interrogated using Sequencher v4.6 (Gene Codes Corporation, MI) to distinguish heterozygous and homozygous samples. Heterozygous sample sets were analyzed for either allelic expression using RT-PCR, methylation-sensitive genotyping or bisulphite PCR, incorporating the polymorphism within the final PCR amplicon so that parental alleles could be distinguished (for primer sequence see S4 Table).

### Quantitative RT-PCR

Expression of the transcripts of interest was analyzed by quantitative real-time RT-PCR with a fluorochrome (SYBR Green) assay and normalized against *RPL19*. Primer sequences are listed in S4 Table. The assays were run in triplicate in 384 well plates in 7900HT Fast Real Time PCR System (Applied Biosystems). Dissociation curves were obtained at the end of each reaction to rule out the presence of primer dimers oabbrevr unexpected DNA species in the reaction. Non-template controls and a calibrator cDNA were included in each assay. Results were analyzed with the SDS 2.3 software (Applied Biosystems). Data analysis was performed using the RQ method and final graphs generated in prism5.

### Bisulphite PCR

Approximately 1 μg DNA was subjected to sodium bisulphite treatment and purified using the EZ DNA methylation-Gold kit (ZYMO, Orange, CA) and was used for all bisulphite PCR analysis. Approximately 2 ul of bisulphite converted DNA was used in each amplification reaction using Immolase Taq polymerase (Bioline) at 45 cycles and the resulting PCR product cloned into pGEM-T easy vector (Promega) for subsequent subcloning and sequencing (for primer sequence see S4 Table).

### Pyrosequencing analysis for methylation quantification

Approximately 50 ng of bisulphite converted DNA was used for pyrosequencing. Standard bisulphite PCR was used to amplify the imprinted DMRs with the exception that one primer was biotinylated (see S4 Table for primer sequences). Previously published primers targeting LINE-1, α-satellites and ALU-Yb8 were used for amplification and sequencing [20]. In all cases the entire biotinylated PCR product (diluted to 40 μl) was mixed with 38 μl of Binding buffer and 2 μl (10 mg/ml) streptavidin-coated polystyrene beads. After washing in 70% ethanol, DNA was denaturated with 50 μl 0.5M NaOH. The single-stranded DNA was hybridized to 40-pmol sequencing primers dissolved in 11 μl annealing buffer at 80°C. For sequencing, forward primers were designed to the complementary strand. The pyrosequencing reaction was carried out on a PyroMark Q96 instrument. The peak heights were determined using Pyro Q-CpG1.0.9 software (Biotage).

### Methylation-sensitive genotyping

Approximately 500 ng of heterozygous genomic DNA was digested with 10 units of *Hpa*II restriction endonuclease for 4 hours at 7°C. The digested DNA was subject to ethanol precipitation and resuspended in a final volume of 20 μl TE or water. Approximately 2 μl of digested DNA was used in each amplification reaction using Bioline Taq polymerase for 40 cycles. The resulting amplicons were sequenced and the sequences traces compared to those obtained for the corresponding undigested DNA template.

### Accession numbers

The Illumina Infinium HumanMethylation450 BeadChip array data has been deposited in the GEO repository and assigned the accession number GSE66247 and GSE52576.

## Supporting Information

S1 Fig Comparison of imprinted methylation profiles in two different RHMs from patient 4. (A) Heatmap of the Infinium probes located within known imprinted DMRs. (B) Heatmap for the Infinium probes mapping to known placenta-specific DMRs. The methylation profiles were confirmed using pyrosequencing for ubiquitous DMRs (C) and placenta-specific DMRs (D). Bisulphite PCR and subcloning confirmation of the methylation difference observed between mole 4 and 4.2 at the *PEG10* DMR. Each circle represents a single CpG dinucleotide on a DNA strand, a methylated cytosine (●) or an unmethylated cytosine (○).
(PDF)

S2 Fig Methylation profiles of four NLRP7 mutated RHMs at imprinted DMRs as determined by pyrosequencing and bisulphite PCR and subcloning. (A) Quantitative pyrosequencing of 16 ubiquitous DMRs and (B) 9 placenta-specific DMRs. The boxplot showing the median methylation (whiskers 5–95% percentile) determined for 15 control placenta samples and the values of RHMs highlighted as shaded circles. (C) Confirmation of the lack-of-methylation at imprinted DMRs by bisulphite PCR and subcloning. Each circle represents a single CpG dinucleotide on a DNA strand, a methylated cytosine (●) or an unmethylated cytosine (○). (D) Cloning for known placenta-specific DMRs.
(PDF)

S3 Fig Quantification of methylation at repeat elements using pyrosequencing. The boxplot showing the median methylation (whiskers 5–95% percentile) determined for 15 control placenta samples and the values of RHMs highlighted.
(PDF)

S4 Fig Confirmation of maternal methylation in term placenta biopsies. The allelic methylation profiles as determined by methylation-sensitive *Hpa*II genotyping in placenta for 18 candidate regions. The asterisk (*) in the sequence traces shows the position of the polymorphic base. The locations of the PCR amplicon are shown for each region.
(PDF)

S5 Fig Strand-specific bisulphite PCR and sequencing of novel placenta-specific DMRs in term placenta biopies. Confirmation of the strand-specific and allelic methylation at seven candidate DMRs associated with the *TTC39A/EPS15*, *THAP3*, *EGR4 RPN1*, *RHOBTB3*, *PURA*, *SNCB*, *THSD7A*, *CCDC71L*, *ARMC3*, *AIFM2*, *FGF8*, *CYB5R2*, *RNF141*, *WIF1*, *RASGRF1* and *SIAH1* genes by bisulphite PCR and subcloning. Each circle represents a single CpG dinucleotide on a DNA strand, a methylated cytosine (●) or an unmethylated cytosine (○) with the letters in the parentheses indicating SNP genotype. The locations of the PCR amplicon are shown for each region.
(PDF)

S6 Fig Allele-specific RT-PCR analysis of candidate placenta-specific imprinted genes. (A) Confirmation of monoallelic expression of *CD83* and paternal expression of *HECW1*, *CCDC71L*, *ST8SIA1*, *RASGRF1*, *CMTM3* and *ZFP90* in term placenta samples. (B) The allele-specific expression analysis of genes flanking known placenta-specific imprinted transcripts. The asterisk (*) in the sequence traces shows the position of the polymorphic base. Biallelic expression of *GLIS3*, *ZC3H12C* and *DNMT1* was also observed in adult somatic tissues. PL = placenta, BR = Brain, KID = Kidney, LY = blood leucocytes.
(PDF)

S7 Fig Genome-wide methylation analysis in blood-derived DNA samples from the four females with recessive *NLRP7* mutations. A heatmap of the Infinium probes located within known imprinted DMRs. As controls for allelic methylation, the profiles of reciprocal uniparental diploidy and four control blood samples are shown.
(PDF)

S1 Table Infinium probe IDs and β values at known and candidate regions. The methylation β values for each of the probes within (A) ubiquitous and (B) known placenta-specific imprinted DMRs. (C) A list of probes, with their corresponding methylation β values, located in candidate imprinted DMRs revealed by the comparison of Infinium methylation profiles of *NLRP7* mutated RHM samples, control placenta and various somatic tissues. (D) The methylation β values for each probe within ubiquitous DMRs for blood-derived DNA from the homozygous *NLRP7* mutated mothers. Each table includes the unique probe ID, map information and corresponding gene name.
(XLSX)

S2 Table The number of heterozygous placenta samples used to determine allelic methylation of novel DMRs using *Hpa*II genotyping. (DOCX)

S3 Table The number of heterozygous placenta samples used to determine allelic expression of novel imprinted transcripts. (DOCX)

S4 Table PCR primer sequences used in this study. (XLSX)

## References

[1] Hoffner L, Surti U. The genetics of gestational trophoblastic disease: a rare complication of pregnancy. Cancer Genet. 2012;205: 63–77. 10.1016/j.cancergen.2012.01.004 22469506

[2] Judson H, Hayward BE, Sheridan E, Bonthron DT. A global disorder of imprinting in the human female germ line. Nature. 2002;416: 539–42. 11932746 10.1038/416539a

[3] Murdoch S, Djuric U, Mazhar B, Seoud M, Khan R, Kuick R, et al Mutations in NALP7 cause recurrent hydatidiform moles and reproductive wastage in humans. Nat Genet. 2006;38: 300–2. 16462743 10.1038/ng1740

[4] Parry DA, Logan CV, Hayward BE, Shires M, Landolsi H, Diggle C, et al Mutations causing familial biparental hydatidiform mole implicate c6orf221 as a possible regulator of genomic imprinting in the human oocyte. Am J Hum Genet. 2011;89: 451–8. 10.1016/j.ajhg.2011.08.002 21885028 PMC3169823

[5] Wang CM, Dixon PH, Decordova S, Hodges MD, Sebire NJ, Ozalp S, et al Identification of 13 novel NLRP7 mutations in 20 families with recurrent hydatidiform mole; missense mutations cluster in the leucine-rich region. J Med Genet. 2009;46: 569–75. 10.1136/jmg.2008.064196 19246479

[6] Dixon PH, Trongwongsa P, Abu-Hayyah S, Ng SH, Akbar SA, Khawaja NP, et al Mutations in NLRP7 are associated with diploid biparental hydatidiform moles, but not androgenetic complete moles. J Med Genet 2012;49: 206–11. 10.1136/jmedgenet-2011-100602 22315435

[7] Reddy R, Akoury E, Phuong Nguyen NM, Abdul-Rahman OA, Dery C, Gupta N, et al Report of four new patients with protein-truncating mutations in C6orf221/KHDC3L and colocalization with NLRP7. Eur J Hum Genet. 2013;21: 957–64. 10.1038/ejhg.2012.274 23232697 PMC3746251

[8] Nguyen NM, Slim R. Genetics and Epigenetics of Recurrent Hydatidiform Moles: Basic Science and Genetic Counselling. Curr Obstet Gynecol Rep. 2014;3: 55–64. 24533231 10.1007/s13669-013-0076-1 PMC3920063

[9] Fisher RA Lavery SA, Carby A, Abu-Hayyeh S, Swingler R, Sebire NJ, Seckl MJ. What a difference an egg makes. Lancet 2011;378: 1974 10.1016/S0140-6736(11)61751-0 22130487

[10] Zhang P, Dixon M, Zucchelli M, Hambiliki F, Levkov L, Hovatta O, et al Expression analysis of the NLRP gene family suggests a role in human preimplantation development. PLoS One. 2008;3: e2755 10.1371/journal.pone.0002755 18648497 PMC2447171

[11] Akoury E, Zhang L, Ao A, Slim R.NLRP7 and KHDC3L, the two maternal-effect proteins responsible for recurrent hydatidiform moles, co-localize to the oocyte cytoskeleton. Hum Reprod. 2015;30: 159–69. 10.1093/humrep/deu291 25358348

[12] Court F, Martin-Trujillo A, Romanelli V, Garin I, Iglesias-Platas I, Salafsky I, et al Genome-wide allelic methylation analysis reveals disease-specific susceptibility to multiple methylation defects in imprinting syndromes. Hum Mutat. 2013;34: 595–602. 10.1002/humu.22276 23335487

[13] Kou YC, Shao L, Peng HH, Rosetta R, del Gaudio D, Wagner AF, et al A recurrent intragenic genomic duplication, other novel mutations in NLRP7 and imprinting defects in recurrent biparental hydatidiform moles. Mol Hum Reprod 2008;14: 33–40. 18039680 10.1093/molehr/gam079

[14] Hayward BE, De Vos M, Talati N, Abdollahi MR, Taylor GR, Meyer E, et al Genetic and epigenetic analysis of recurrent hydatidiform mole. Hum Mutat. 2009;30: E629–39 10.1002/humu.20993 19309689

[15] Ferguson-Smith AC. Genomic imprinting: the emergence of an epigenetic paradigm. Nat Rev Genet. 2011;12: 565–75. 10.1038/nrg3032 21765458

[16] Duéñez-Guzmán EA, Haig D. The evolution of reproduction-related NLRP genes. J Mol Evol. 2014;78: 194–201. 10.1007/s00239-014-9614-3 24615281

[17] Meyer E, Lim D, Pasha S, Tee LJ, Rahman F, Yates JR, et al Germline mutation in NLRP2 (NALP2) in a familial imprinting disorder (Beckwith-Wiedemann Syndrome). PLoS Genet. 2009;5: e1000423 10.1371/journal.pgen.1000423 19300480 PMC2650258

[18] Court F, Tayama C, Romanelli V, Martin-Trujillo A, Iglesias-Platas I, Okamura K, et al Genome-wide parent-of-origin DNA methylation analysis reveals the intricacies of human imprinting and suggests a germline methylation-independent mechanism of establishment. Genome Res. 2014;24: 554–69. 10.1101/gr.164913.113 24402520 PMC3975056

[19] Okae H, Chiba H, Hiura H, Hamada H, Sato A, Utsunomiya T, et al Genome-wide analysis of DNA methylation dynamics during early human development. PloS Genet. 2014;10: e1004868 10.1371/journal.pgen.1004868 25501653 PMC4263407

[20] Camprubí C, Iglesias-Platas I, Martin-Trujillo A, Salvador-Alarcon C, Rodriguez MA, Barredo DR, et al Stability of genomic imprinting and gestational-age dynamic methylation in complicated pregnancies conceived following assisted reproductive technologies. Biol Reprod. 2013;89: 50 10.1095/biolreprod.113.108456 23884645

[21] Metsalu T, Viltrop T, Tiirats A, Rajashekar B, Reimann E, Kõks S, et al Using RNA sequencing for identifying gene imprinting and random monoallelic expression in human placenta. Epigenetics. 2014;9: 1397–409. 10.4161/15592294.2014.970052 25437054 PMC4623103

[22] Pozharny Y, Lambertini L, Ma Y, Ferrara L, Litton CG, Diplas A, et al Genomic loss of imprinting in first-trimester human placenta. Am J Obstet Gynecol. 2010;202: 391.e1–8. 20350649 10.1016/j.ajog.2010.01.039

[23] Kobayashi H, Yanagisawa E, Sakashita A, Sugawara N, Kumakura S, Ogawa H, et al Epigenetic and transcriptional features of the novel human imprinted lncRNA *GPR1AS* suggest it is a functional orthology to mouse *Zdbf2linc* . Epigenetics. 2013;8: 635–45. 10.4161/epi.24887 23764515 PMC3857343

[24] Duffié R, Ajjan S, Greenberg M, Zamudio N, Secamilla del Arenal M, Iranzo J, et al The Gpr1/Zdbf2 locus provides new paradigms for transient and dynamic genomic imprinting in mammals. Genes & Dev. 2014;28: 463–78. 24589776 10.1101/gad.232058.113 PMC3950344

[25] Yuen RK, Jiang R, Peñaherrera MS, McFadden DE, Robinson WP (2011) Genome-wide mapping of imprinted differentially methylated regions by DNA methylation profiling of human placentas from triploidies. Epigenetics Chromatin 4(1):10 10.1186/1756-8935-4-10 21749726 PMC3154142

[26] Herse F, Lamarca B, Hubel CA, Kaartokallio T, Lokki AI, Ekholm E, et al Cytochrome P450 subfamily 2J polypeptide 2 expression and circulating epoxyeicosatrienoic metabolites in preeclampsia. Circulation 2012;126: 2990–9. 10.1161/CIRCULATIONAHA.112.127340 23155181 PMC3543781

[27] Kuo MW, Wang CH, Wu HC, Chang SJ, Chuang YJ. Soluble THSD7A is an N-glycoprotein that promotes endothelial cell migration and tube formation in angiogenesis. PLoS One. 2011;6: e29000 10.1371/journal.pone.0029000 22194972 PMC3237571

[28] Noguer-Dance M, Abu-Amero S, Al-Khtib M, Lefèvre A, Coullin P, et al The primate-specific microRNA gene cluster (C19MC) is imprinted in the placenta. Hum Mol Genet 2010;19: 3566–82. 10.1093/hmg/ddq272 20610438

[29] Xie L, Mouillet JF, Chu T, Parks WT, Sadovsky E, Knöfler M, et al C19MC MicroRNAs Regulate the Migration of Human Trophoblasts. Endocrinology. 2014;155: 4975–85. 10.1210/en.2014-1501 25211593 PMC4239420

[30] Guo H, Zhu P, Yan L, Li R, Hu B, Lian Y, et al The DNA methylation landscape of human early embryos. Nature. 2014;511: 606–10. 10.1038/nature13544 25079557

[31] Smith ZD, Chan MM, Humm KC, Karnik R, Mekhoubad S, Regev A, et al (2014) DNA methylation dynamics of the human preimplantation embryo. Nature 2014;511: 611–5. 10.1038/nature13581 25079558 PMC4178976

[32] Li X, Ito M, Zhou F, Youngson N, Zuo X, Leder P, et al A maternal-zygotic effect gene, Zfp57, maintains both maternal and paternal imprints. Dev Cell. 2008;15: 547–57. 10.1016/j.devcel.2008.08.014 18854139 PMC2593089

[33] Nakamura T, Arai Y, Umehara H, Masuhara M, Kimura T, Taniguchi H, et al PGC7/Stella protects against DNA demethylation in early embryogenesis. Nat Cell Biol. 2007;9: 64–71. 17143267 10.1038/ncb1519

[34] Wossidlo M, Nakamura T, Lepikhov K, Marques CJ, Zakhartchenko V, Boiani M, et al 5-Hydroxymethylcytosine in the mammalian zygote is linked with epigenetic reprogramming. Nat Commun. 2011;2: 241 10.1038/ncomms1240 21407207

[35] Petrussa L, Van de Velde H, De Rycke M. Dynamic regulation of DNA methyltransferases in human oocytes and preimplantation embryos after assisted reproductive technologies. Mol Hum Reprod. 2014;20: 861–74. 10.1093/molehr/gau049 24994815

[36] Mahadevan S, Wen S, Wan YW, Peng HH, Otta S, Liu Z, et al NLRP7 affects trophoblast lineage differentiation, binds to overexpressed YY1 and alters CpG methylation. Hum Mol Genet. 2014;23: 706–16. 10.1093/hmg/ddt457 24105472 PMC3888260

[37] Singer H, Biswas A, Nuesgen N, Oldenburg J, El-Maarri O. NLRP7, Involved in hydatidiform molar pregnancy (HYDM1), interacts with the transcriptional repressor ZBTB16. PLoS One. 2015: e0130416 10.1371/journal.pone.0130416 26121690 PMC4488268

[38] Messaed C, Akoury E, Djuric U, Zeng J, Saleh M, Gilbert L, et al NLRP7, a nucleotide oligomerization domain-like receptor protein, is required for normal cytokine secretion and co-localizes with Golgi and the microtubule-organizing center. J Biol Chem. 2011;286: 43313–23. 10.1074/jbc.M111.306191 22025618 PMC3234874

[39] Caillaud M, Duchamp G, Gérard N. In vivo effect of interleukin-1beta and interleukin-1RA on oocyte cytoplasmic maturation, ovulation, and early embryonic development in the mare. Reprod Biol Endocrinol. 2005;3: 26 15972098 10.1186/1477-7827-3-26 PMC1182395

[40] Gkountela S, Li Z, Vincent JJ, Zhang KX, Chen A, Pellegrini M, et al The ontogeny of cKIT+ human primordial germ cells proves to be a resource for human germ line reprogramming, imprint erasure and in vitro differentiation. Nat Cell Biol. 2013;15: 113–22. 10.1038/ncb2638 23242216 PMC3786872

[41] Nakabayashi K, Trujillo AM, Tayama C, Camprubi C, Yoshida W, Lapunzina P, et al Methylation screening of reciprocal genome-wide UPDs identifies novel human-specific imprinted genes. Hum Mol Genet. 2011;20: 3188–97. 10.1093/hmg/ddr224 21593219

