# Supplementary figures and images for: Absence of Maternal Methylation in Biparental Hydatidiform Moles from Women with NLRP7 Maternal-Effect Mutations Reveals Widespread Placenta-Specific Imprinting
Source: PLoS Genet. 2015 Nov 6;11(11):e1005644. doi: 10.1371/journal.pgen.1005644 (PMC4636177; doi:10.1371/journal.pgen.1005644)

# S1\_Fig

**A**

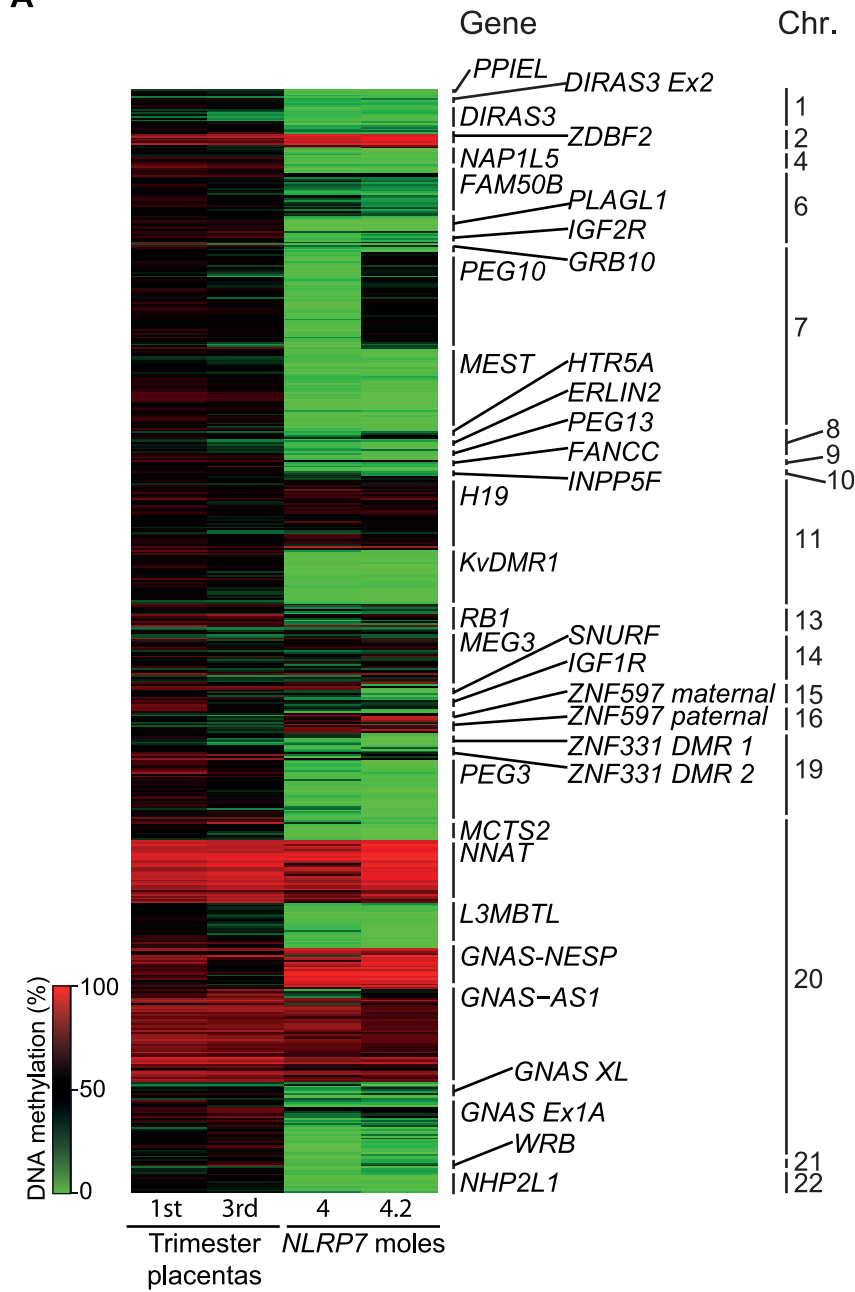

**B**

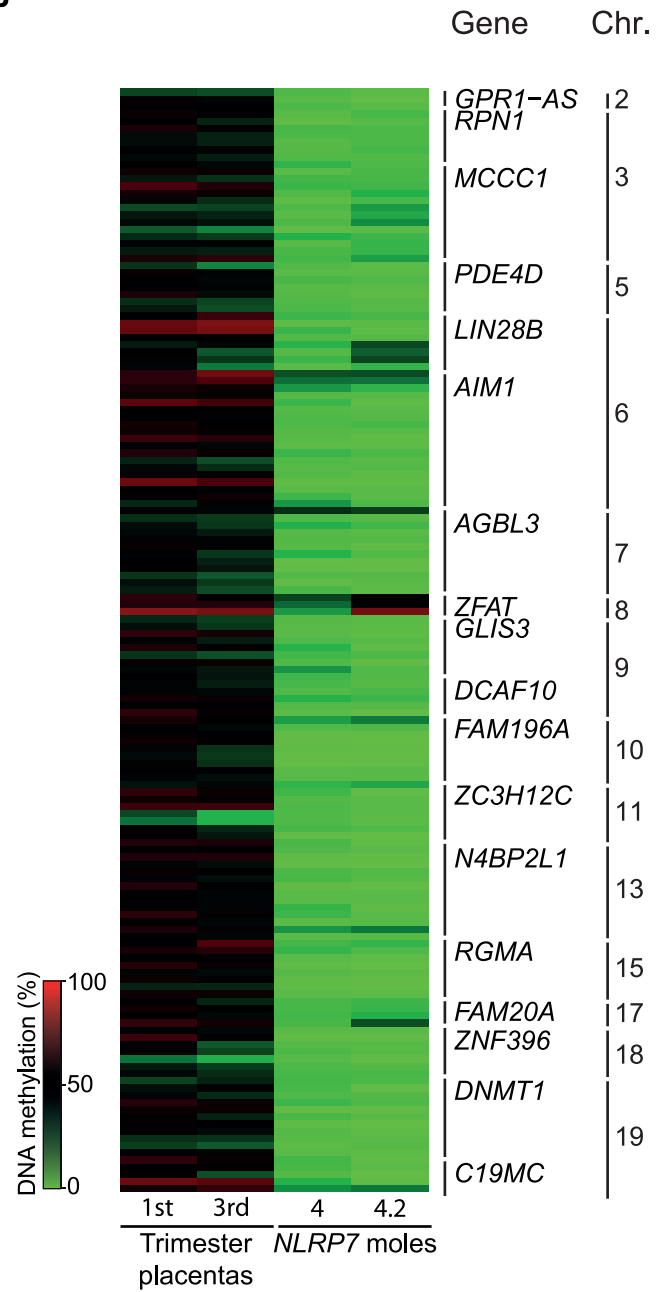

**C**

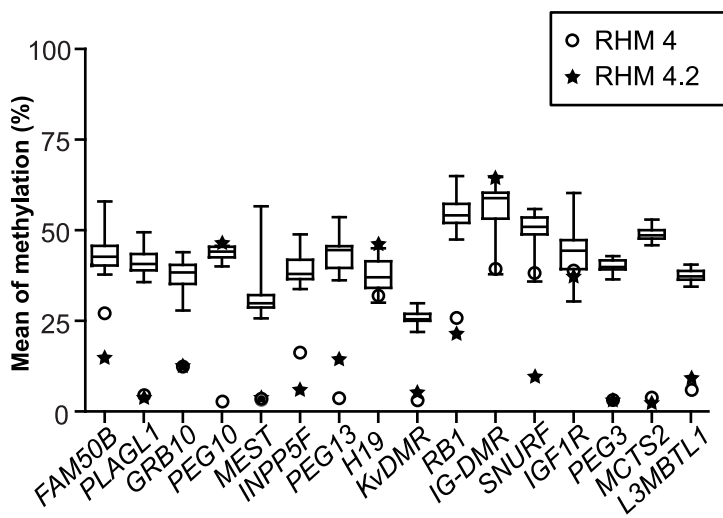

**D**

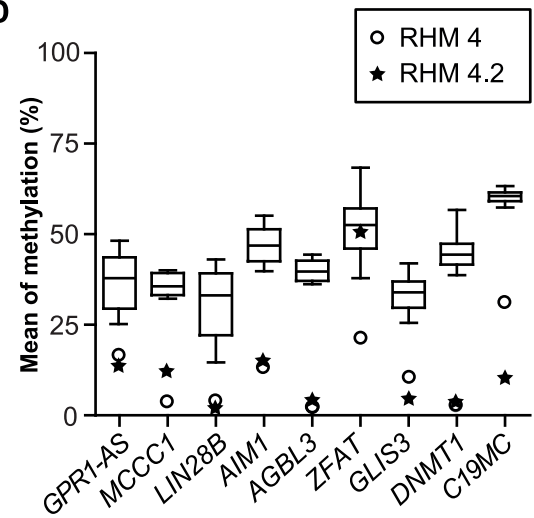

**E**

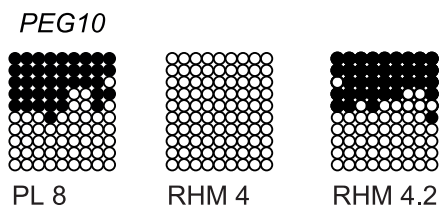

Supplement: S1 Fig — (A) Heatmap of the Infinium probes located within known imprinted DMRs. (B) Heatmap for the Infinium probes mapping to known placenta-specific DMRs. The methylation profiles were confirmed using pyrosequencing for ubiquitous DMRs (C) and placenta-specific DMRs (D). Bisulphite PCR and subcloning confirmation of the methylation difference observed between mole 4 and 4.2 at the PEG10 DMR. Each circle represents a single CpG dinucleotide on a DNA strand, a methylated cytosine (●) or an unmethylated cytosine (○). (PDF) [file pgen.1005644.s001.pdf]

S2\_Fig

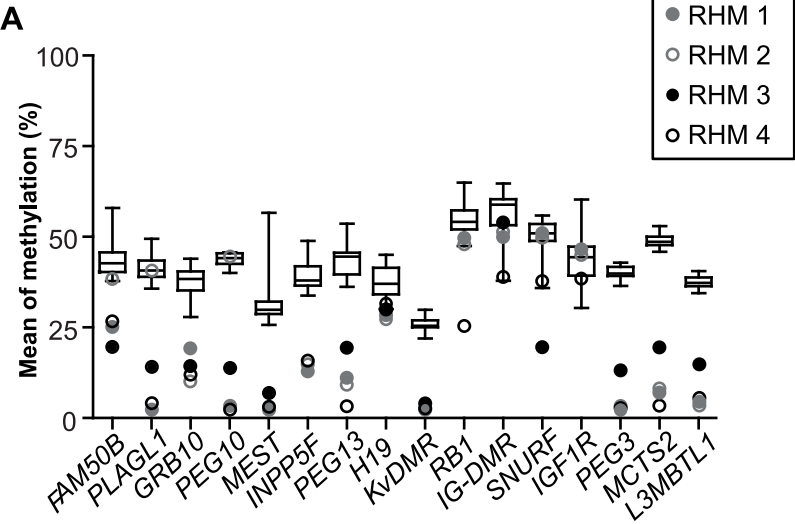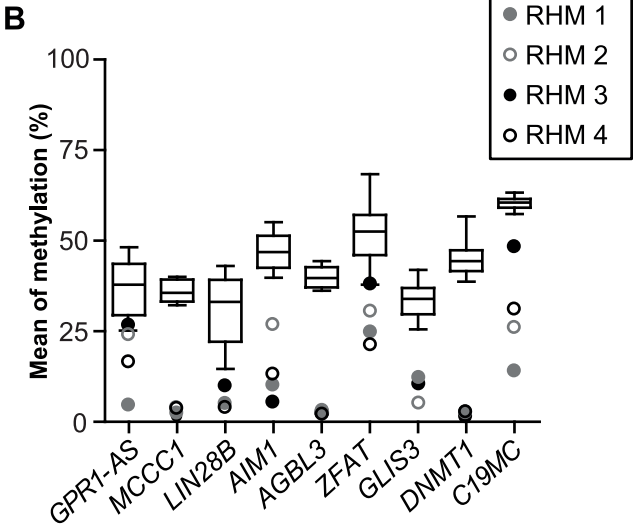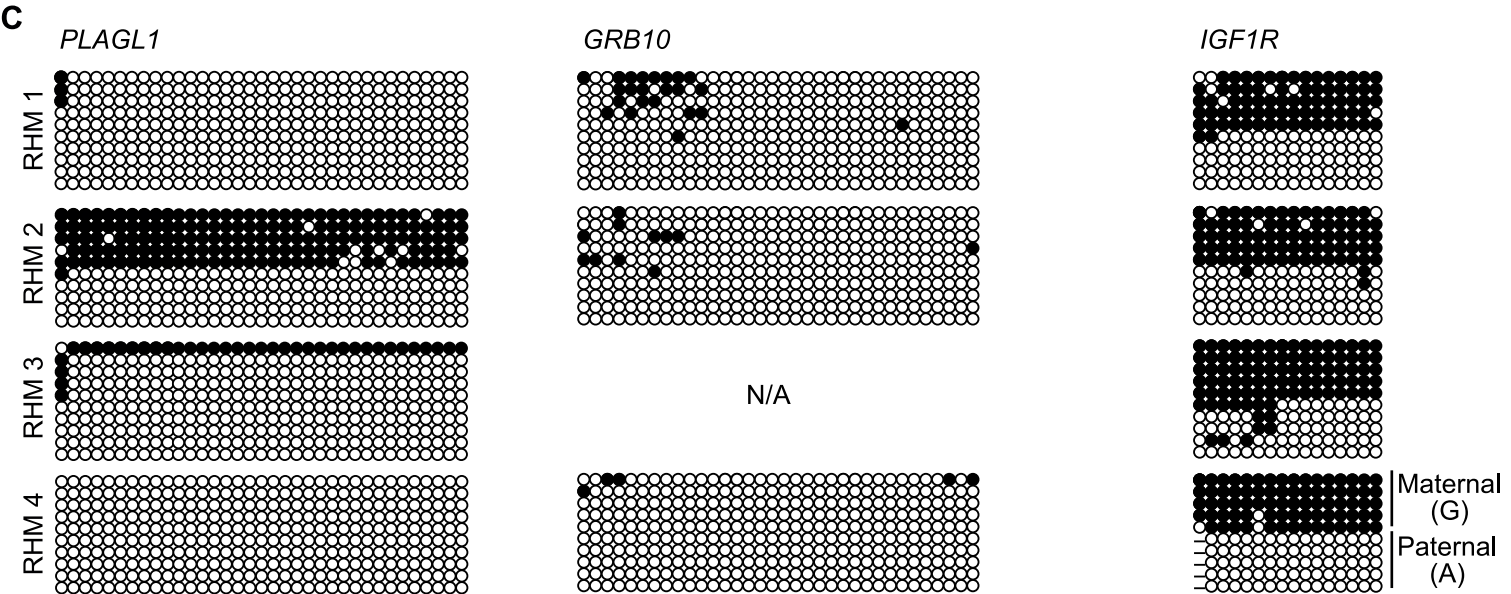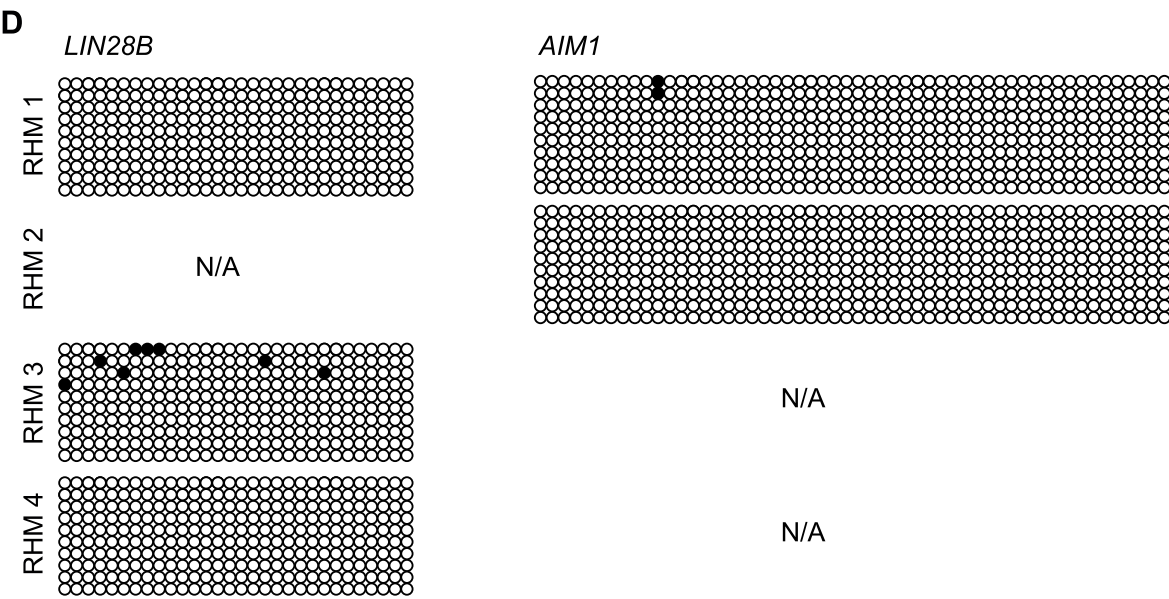

Supplement: S2 Fig — (A) Quantitative pyrosequencing of 16 ubiquitous DMRs and (B) 9 placenta-specific DMRs. The boxplot showing the median methylation (whiskers 5–95% percentile) determined for 15 control placenta samples and the values of RHMs highlighted as shaded circles. (C) Confirmation of the lack-of-methylation at imprinted DMRs by bisulphite PCR and subcloning. Each circle represents a single CpG dinucleotide on a DNA strand, a methylated cytosine (●) or an unmethylated cytosine (○). (D) Cloning for known placenta-specific DMRs. (PDF) [file pgen.1005644.s002.pdf]

S3\_Fig

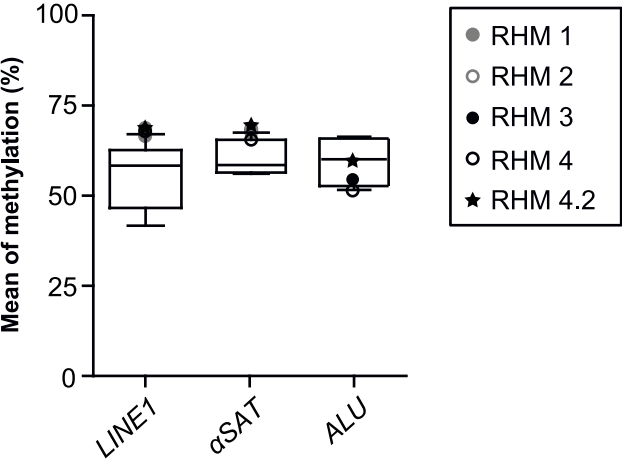

Supplement: S3 Fig — The boxplot showing the median methylation (whiskers 5–95% percentile) determined for 15 control placenta samples and the values of RHMs highlighted. (PDF) [file pgen.1005644.s003.pdf]

S4\_Fig

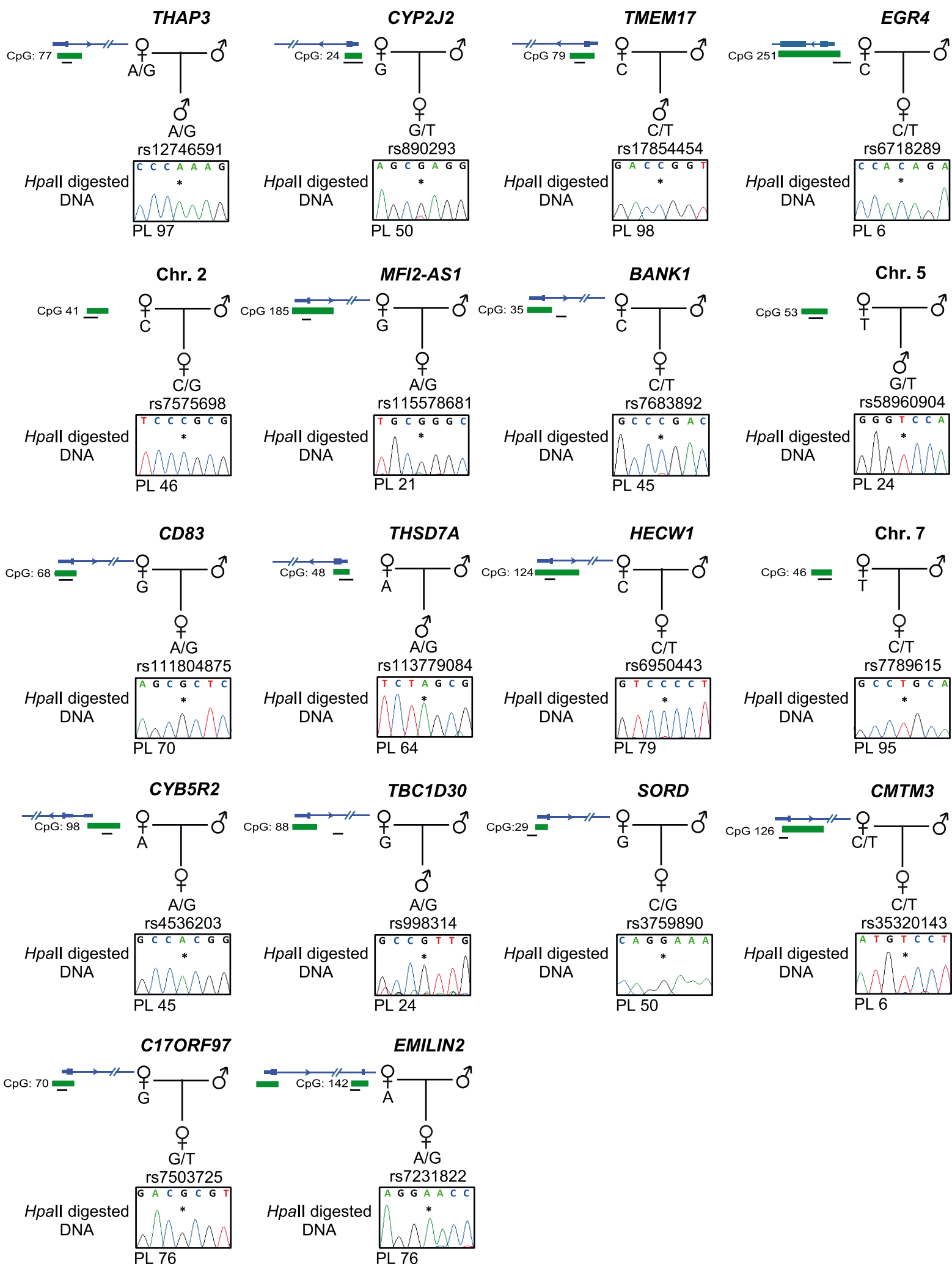

Supplement: S4 Fig — The allelic methylation profiles as determined by methylation-sensitive HpaII genotyping in placenta for 18 candidate regions. The asterisk (*) in the sequence traces shows the position of the polymorphic base. The locations of the PCR amplicon are shown for each region. (PDF) [file pgen.1005644.s004.pdf]

S5\_Fig

*TTC39A/EPS15*

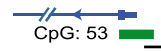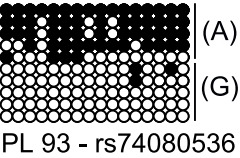

*THAP3*

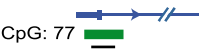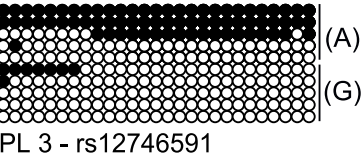

*EGR4*

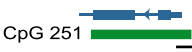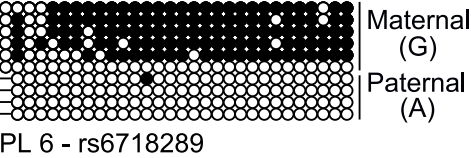

*RPN1*

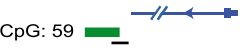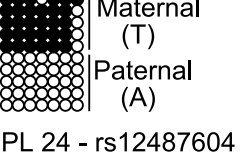

*RHOBTB3*

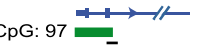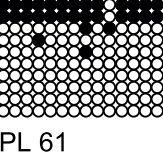

*PURA*

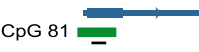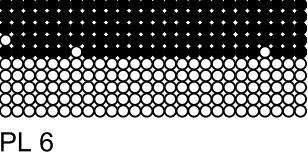

*SNCB*

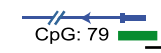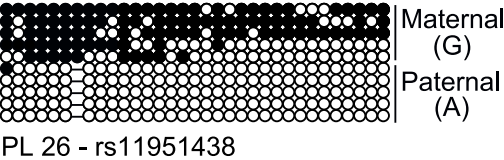

*THSD7A*

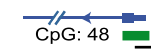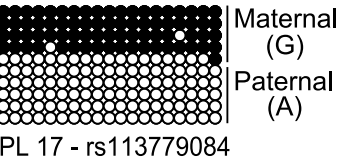

*CCDC71L*

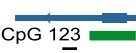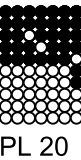

*ARMC3*

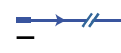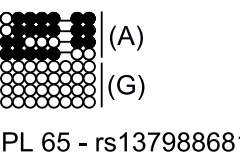

*AIFM2*

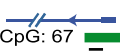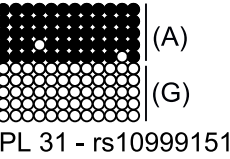

*FGF8*

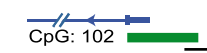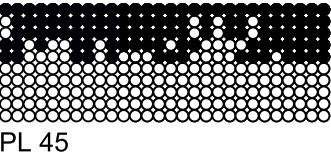

*CYB5R2*

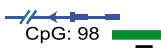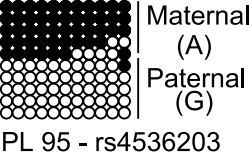

*RNF141*

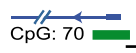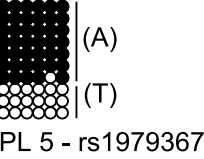

*WIF1*

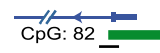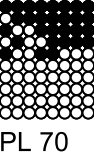

*RASGRF1*

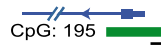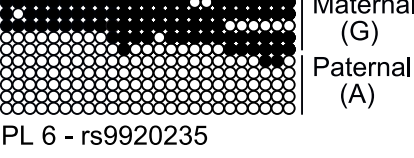

*SIAH1*

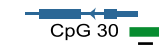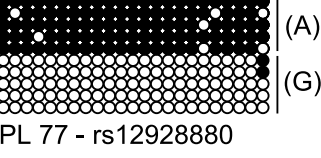

Supplement: S5 Fig — Confirmation of the strand-specific and allelic methylation at seven candidate DMRs associated with the TTC39A/EPS15, THAP3, EGR4 RPN1, RHOBTB3, PURA, SNCB, THSD7A, CCDC71L, ARMC3, AIFM2, FGF8, CYB5R2, RNF141, WIF1, RASGRF1 and SIAH1 genes by bisulphite PCR and subcloning. Each circle represents a single CpG dinucleotide on a DNA strand, a methylated cytosine (●) or an unmethylated cytosine (○) with the letters in the parentheses indicating SNP genotype. The locations of the PCR amplicon are shown for each region. (PDF) [file pgen.1005644.s005.pdf]

# S6\_Fig

A

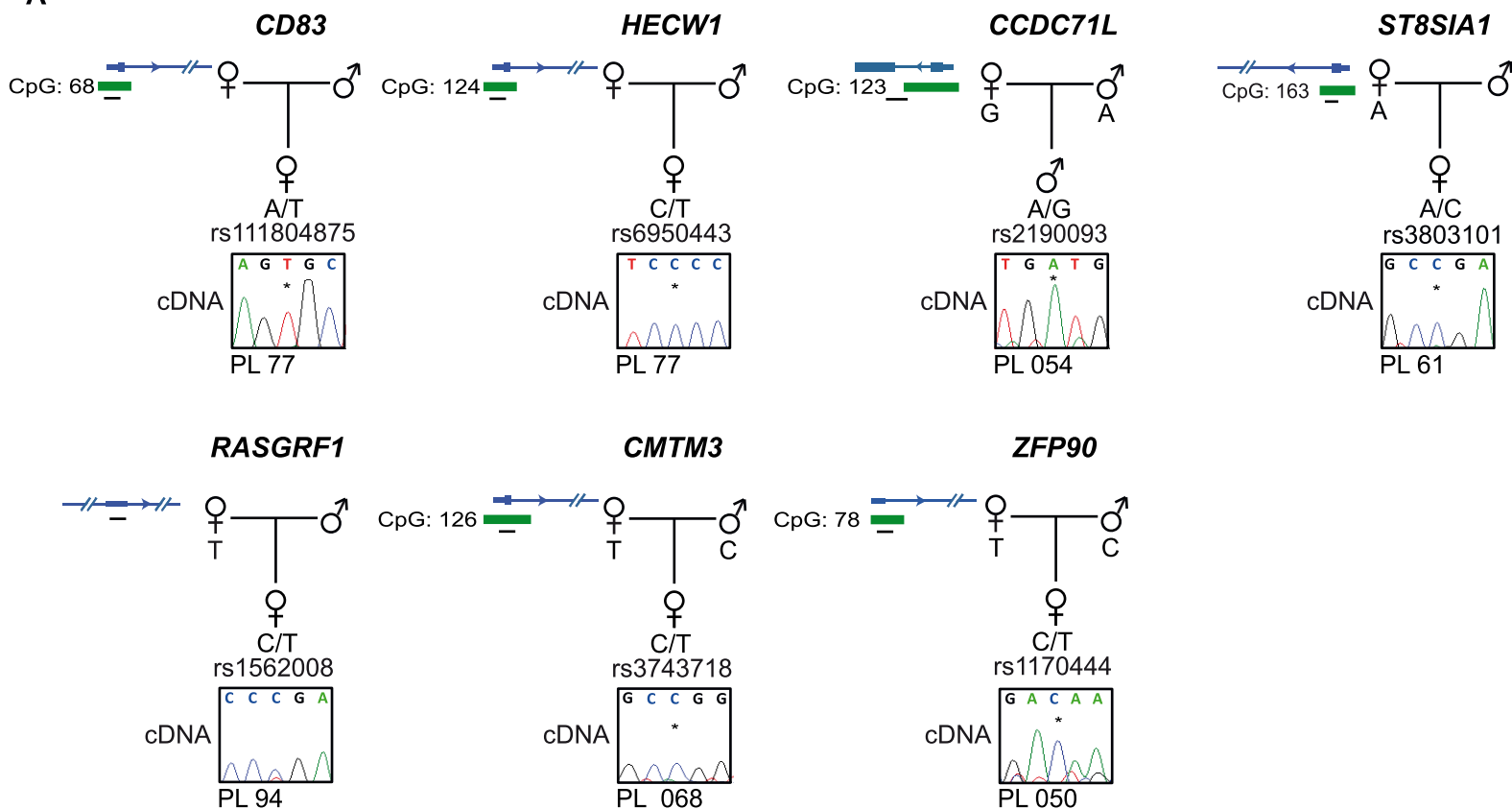

B

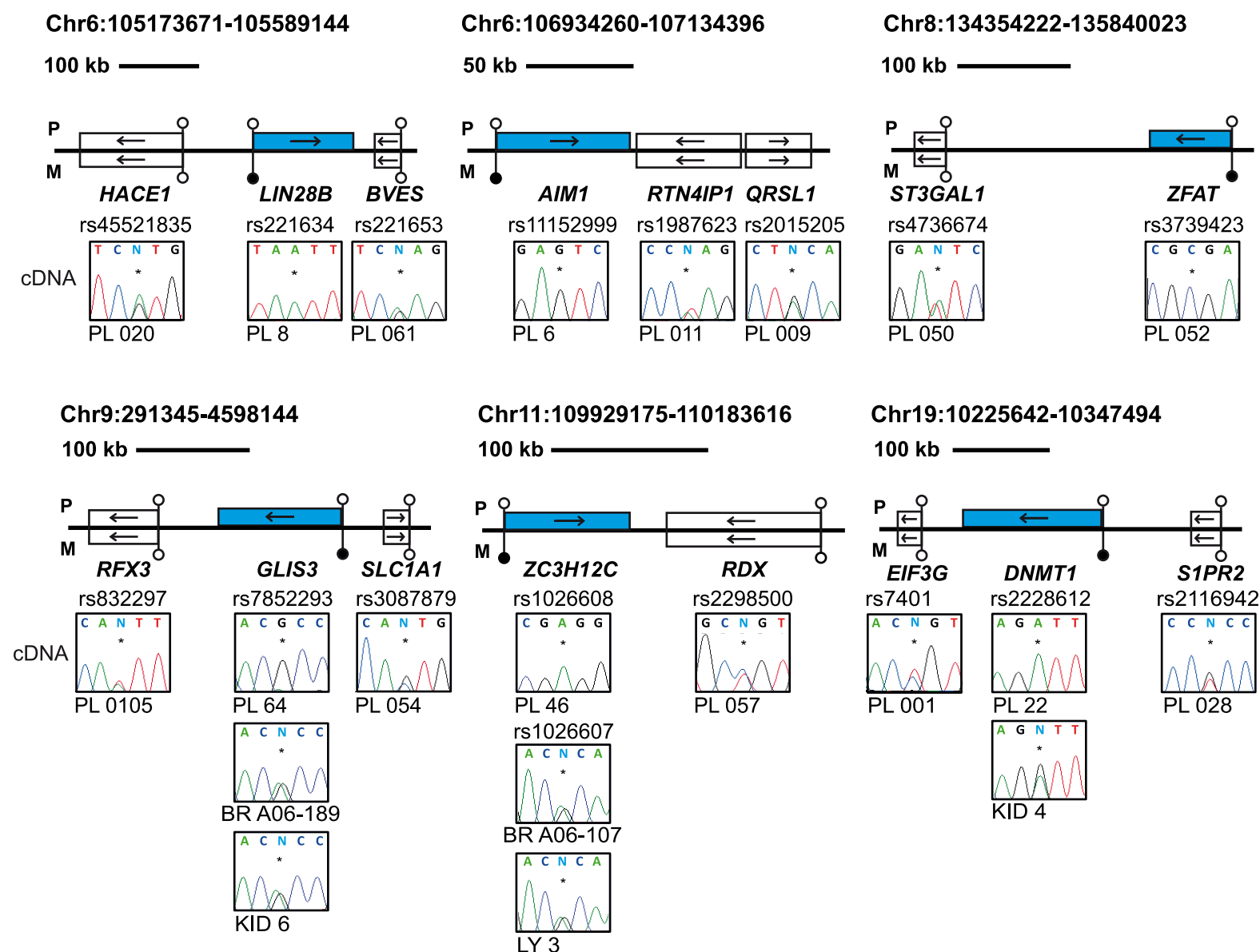

Supplement: S6 Fig — (A) Confirmation of monoallelic expression of CD83 and paternal expression of HECW1, CCDC71L, ST8SIA1, RASGRF1, CMTM3 and ZFP90 in term placenta samples. (B) The allele-specific expression analysis of genes flanking known placenta-specific imprinted transcripts. The asterisk (*) in the sequence traces shows the position of the polymorphic base. Biallelic expression of GLIS3, ZC3H12C and DNMT1 was also observed in adult somatic tissues. PL = placenta, BR = Brain, KID = Kidney, LY = blood leucocytes. (PDF) [file pgen.1005644.s006.pdf]

S7\_Fig

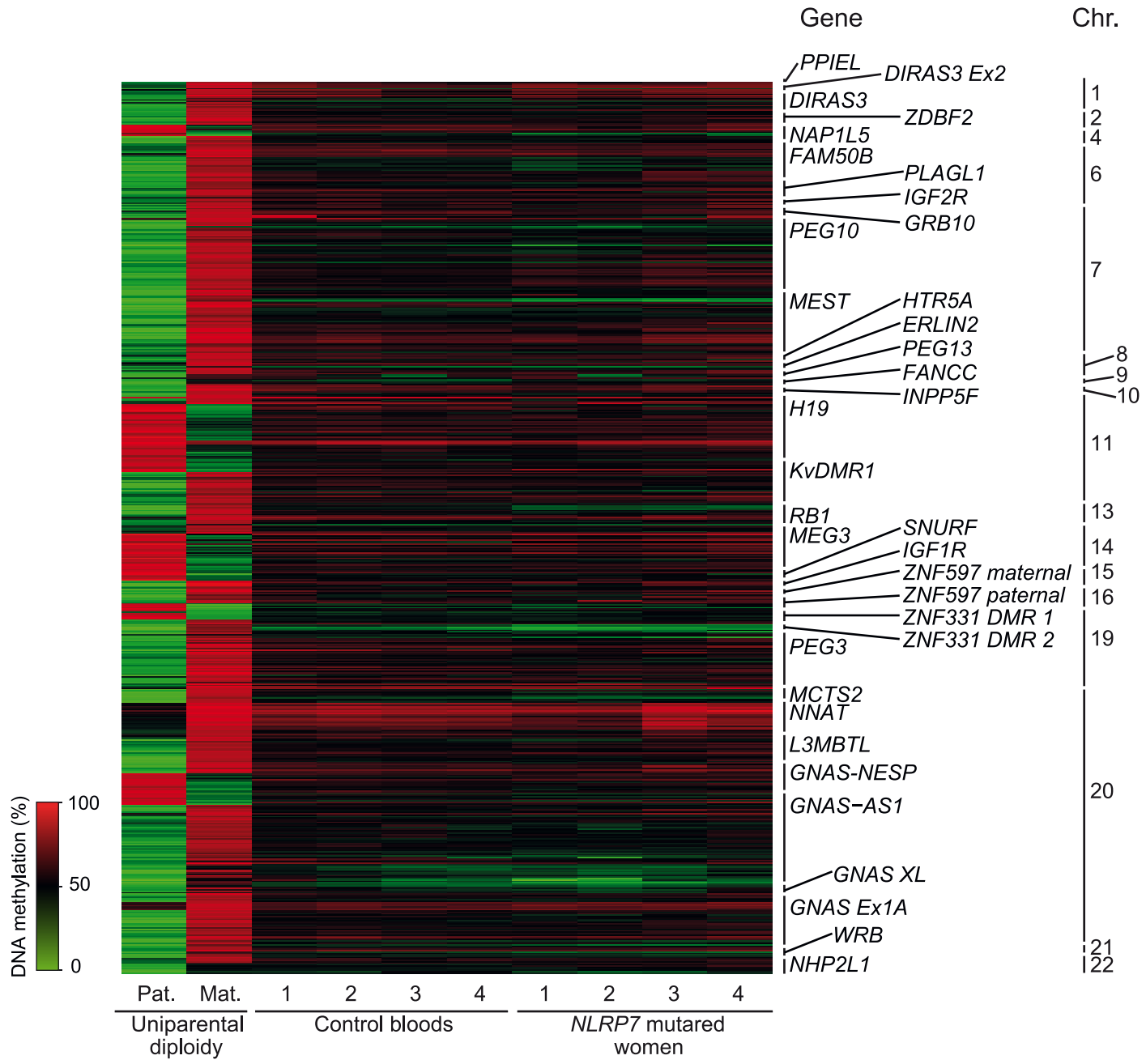

Supplement: S7 Fig — A heatmap of the Infinium probes located within known imprinted DMRs. As controls for allelic methylation, the profiles of reciprocal uniparental diploidy and four control blood samples are shown. (PDF) [file pgen.1005644.s007.pdf]
