# Supplementary material for: Absence of Maternal Methylation in Biparental Hydatidiform Moles from Women with NLRP7 Maternal-Effect Mutations Reveals Widespread Placenta-Specific Imprinting
Source: PLoS Genet. 2015 Nov 6;11(11):e1005644. doi: 10.1371/journal.pgen.1005644 (PMC4636177; doi:10.1371/journal.pgen.1005644)
Supplement: S2 Table — (DOCX) [file pgen.1005644.s009.docx]

**S2_Table**

| **Gene** | **Number of heterozygous samples assessed by methylation-sensitive genotyping PCR** |
| --- | --- |
| *THAP3* | 3 uninformative monoallelic placenta samples |
| *CYP2J2* | 2 maternal, 3 uninformative monoallelic and 2 biallelic placenta samples |
| *TMEM17* | 2 maternal and 9 uninformative monoallelic placenta samples |
| *EGR4* | 1 maternal and 2 uninformative monoallalic placenta samples |
| *Chr.2* | 1 maternal and 4 biallelic placenta samples |
| *MFI2-AS1* | 2 maternal, 1 uninformative monoallelic and 2 biallelic placenta samples |
| *BANK1* | 2 maternal and 5 uninformative monoallelic placenta samples |
| *Chr.5* | 4 maternal and 5 uninformative monoallelic placenta samples. |
| *CD83* | 2 maternal and 3 uninformative monoallelic placenta samples |
| *THSD7A* | 2 maternal and 6 uninformative monoallelic placenta samples |
| *SCIN* | 1 maternal and 3 uninformative monoallelic placenta samples |
| *HECW1* | 3 maternal and 3 uninformative monoallelic placenta samples |
| *Chr.7* | 2 maternal, 5 uninformative monoallelic and 3 biallelic placenta samples |
| *CYB5R2* | 2 maternal and 1 uninformative monoallelic placenta samples |
| *ST8SIA1* | 1 maternal and 5 uninformative monoallelic placenta samples |
| *TBC1D30* | 2 maternal, 5 uninformative monoallelic and 3 biallelic placenta samples |
| *SORD* | 1 maternal placenta samples |
| *CMTM3* | 3 uninformative monoallelic placenta samples |
| *C17ORF97* | 1 maternal, 1 uninformative monoallelic and 1 biallelic placenta samples |
| *EMILIN2* | 3 maternal and 5 uninformative monoallelic placenta samples |
| *CABIN1* | 2 maternal and 3 uninformative monoallelic placenta samples |

The number of heterozygous placenta samples used to determine allelic methylation of novel imprinted DMRs.
