# Supplementary material for: Absence of Maternal Methylation in Biparental Hydatidiform Moles from Women with NLRP7 Maternal-Effect Mutations Reveals Widespread Placenta-Specific Imprinting
Source: PLoS Genet. 2015 Nov 6;11(11):e1005644. doi: 10.1371/journal.pgen.1005644 (PMC4636177; doi:10.1371/journal.pgen.1005644)
Supplement: S3 Table — (DOCX) [file pgen.1005644.s010.docx]

**S3_Table**

| **Gene** | **Number of heterozygous samples assessed by allelic RT-PCR** |
| --- | --- |
| *ZNF396* | 4 paternally expressed and 1 biallelic |
| *AIM1* | 5 paternally expressed, 2 monoallelically expressed and 1 biallelic |
| *MCCC1* | 2 paternally expressed |
| *AGBL3* | 7 paternally expressed |
| *LIN28B* | 5 paternally expressed |
| *ZFAT* | 5 paternally expressed |
| *GLIS3* | 2 paternally expressed and 1 monoallelically expressed |
| *ZC3H12C* | 1 paternally expressed |
| *DNMT1* | 17 paternally expressed and 3 monoallelically expressed |
| *GPR1-AS1* | 3 paternally expressed and 5 monoallelically expressed |
| *ZBDF2* | 8 monoallelically expressed |
| *SCIN* | 6 paternal, 2 monoallelically expressed from the unmethylated allele and 3 biallelic |
| *RASGRF1* | 2 paternally expressed and 2 biallelic |
| *RHOBTB3* | 2 paternal and 8 monoallelically expressed |
| *CMTM3* | 1 paternal, 3 monoallelically expressed from the unmethylated allele and 2 biallelic |
| *CD83* | 1 monoallelically expressed from the unmethylated allele |
| *ST8SIA1* | 1 paternal, 3 monoallelically expressed from the unmethylated allele |
| *HECW1* | 1 paternally expressed, 1 monoallelically expressed from the unmethylated allele and 1 biallelically expressed |
| *ZFP90* | 3 paternally expressed and 2 biallelically expressed |
| *CCDC71L* | 2 paternally expressed and 2 biallelically expressed |
| *AIFM2* | 6 biallelically expressed |
| *CABIN1* | 6 biallelically expressed |
| *TBC1D30* | 3 biallelically expressed |
| *CYB5R2* | 5 biallelically expressed |
| *ADAM23* | 11 paternally expressed |
| *S1PR2* | 6 biallelically expressed |
| *LAMP3* | 6 biallelically expressed |
| *DCUN1D1* | 6 biallelically expressed |
| *RFX3* | 1 biallelically expressed |
| *SLC1A1* | 5 biallelically expressed |
| *RDX* | 6 biallelically expressed |
| *WDR91* | 6 biallelically expressed |
| *EIF3G* | 6 biallelically expressed |
| *RTN4IP1* | 6 biallelically expressed |
| *QRSL1* | 6 biallelically expressed |
| *HACE1* | 5 biallelically expressed |
| *BVES* | 6 biallelically expressed |
| *ST3GAL1* | 5 biallelically expressed |
| *ZNF24* | 5 biallelically expressed |
| *INO80C* | 4 biallelically expressed |
| *VWDE* | *Not expressed* |
| *ARL4A* | 6 biallelically expressed |
| *GLRX* | 6 biallelically expressed |
| *SPATA9* | *Not expressed* |
| *CALD1* | 5 biallelically expressed |
| *TMEM140* | 4 biallelically expressed |

The number of heterozygous placenta samples used to determine allelic RT-PCR.
